# Definition of the σ^W^ Regulon of *Bacillus subtilis* in the Absence of Stress

**DOI:** 10.1371/journal.pone.0048471

**Published:** 2012-11-14

**Authors:** Jessica C. Zweers, Pierre Nicolas, Thomas Wiegert, Jan Maarten van Dijl, Emma L. Denham

**Affiliations:** 1 Department of Medical Microbiology, University of Groningen, University Medical Center Groningen, Groningen, The Netherlands; 2 INRA, UR1077, Mathématique Informatique et Génome, Jouy-en-Josas, France; 3 Hochschule Zittau/Görlitz, FN/Biotechnologie, Zittau, Germany; Loyola University Medical Center, United States of America

## Abstract

Bacteria employ extracytoplasmic function (ECF) sigma factors for their responses to environmental stresses. Despite intensive research, the molecular dissection of ECF sigma factor regulons has remained a major challenge due to overlaps in the ECF sigma factor-regulated genes and the stimuli that activate the different ECF sigma factors. Here we have employed tiling arrays to single out the ECF σ^W^ regulon of the Gram-positive bacterium *Bacillus subtilis* from the overlapping ECF σ^X^, σ^Y^, and σ^M^ regulons. For this purpose, we profiled the transcriptome of a *B. subtilis sigW* mutant under non-stress conditions to select candidate genes that are strictly σ^W^-regulated. Under these conditions, σ^W^ exhibits a basal level of activity. Subsequently, we verified the σ^W^-dependency of candidate genes by comparing their transcript profiles to transcriptome data obtained with the parental *B. subtilis* strain 168 grown under 104 different conditions, including relevant stress conditions, such as salt shock. In addition, we investigated the transcriptomes of *rasP* or *prsW* mutant strains that lack the proteases involved in the degradation of the σ^W^ anti-sigma factor RsiW and subsequent activation of the σ^W^-regulon. Taken together, our studies identify 89 genes as being strictly σ^W^-regulated, including several genes for non-coding RNAs. The effects of *rasP* or *prsW* mutations on the expression of σ^W^-dependent genes were relatively mild, which implies that σ^W^-dependent transcription under non-stress conditions is not strictly related to RasP and PrsW. Lastly, we show that the pleiotropic phenotype of *rasP* mutant cells, which have defects in competence development, protein secretion and membrane protein production, is not mirrored in the transcript profile of these cells. This implies that RasP is not only important for transcriptional regulation via σ^W^, but that this membrane protease also exerts other important post-transcriptional regulatory functions.

## Introduction

Extracytoplasmic function (ECF) sigma factors enable bacteria to respond adequately to harsh and stressful environmental conditions. The numbers of ECF sigma factors vary among different bacteria. While some bacteria (e.g. *Mycoplasma genitalium*) have no ECF sigma factors, other bacteria can contain over 50 (*Streptomyces coelicolor*). In most cases however, only a limited number of ECF sigma factors are present. For example, *Escherichia coli* produces 2, and *Bacillus subtilis* 7 [23]. In non-stressed cells, these sigma factors are usually inhibited by binding to a specific anti-sigma factor [49]. For several anti-sigma factors it has been shown that specific extracellular stresses trigger their regulated intramembrane proteolysis (RIP) by site-1 and site-2 proteases in the membrane [22], [24], [29], [44]. Specifically, the site-1 protease clips in the extracytoplasmic part of the anti-sigma factor and renders it a substrate for the intramembrane cleaving site-2 protease. This results in the release of the anti-sigma factor/sigma factor complex into the cytoplasm, where the anti-sigma factor is further degraded and the sigma factor can then redirect transcription [10], [21], [23], [27], [29]. Attempts to accurately define each of the ECF sigma factor regulons in organisms with multiple ECF sigma factors have been complicated by partial overlaps that exist both for the binding sites recognized by these sigma factors and the stimuli that activate them. This is very clearly illustrated by studies on the σ^W^, σ^X^, σ^Y^ and σ^M^ sigma factors and their regulons in *B. subtilis* [8], [9], [14], [25], [26], [33], [34], [45]. To single out the individual ECF sigma factor regulons is challenging, which is underscored by a recent classification of the promoters of *B. subtilis* based on an unsupervised algorithm [35]. This approach, which involved transcript profiling across 104 different conditions, only allowed the identification of a global ECF regulon, while the individual σ^W^, σ^X^, σ^Y^ and σ^M^ regulons remained undefined.

The σ^W^ regulon is among the three best-studied ECF sigma factor regulons in *B. subtilis*. This regulon is induced in response to cell envelope stress caused by antibiotics, alkaline shock and salt shock [8], [9], [18], [31], [38], [39], [43], [48]. The anti-sigma factor of σ^W^, RsiW, is cleaved by the site-1 protease PrsW and the site-2 protease RasP [12], [15], [21], [42], [49]. Consistent with the requirement of PrsW for RsiW degradation, *prsW* mutant cells have a phenotype that is very similar to the phenotype of *sigW* mutant cells. In contrast, deletion of the *rasP* gene causes a pleiotropic phenotype including defects in the development of competence for genetic transformation and protein secretion [20], [41]. Although transcriptional analyses with *sigW* mutant cells were previously performed [8], a detailed comparison of the effects of a *sigW* mutation with those of *prsW* or *rasP* mutations on genome-wide transcription has not yet been documented. Additionally, in the previous transcriptional analyses of the *sigW* deletion strain, non-coding RNAs (ncRNAs) were not included. Therefore, the present studies were aimed at defining the strictly σ^W^-regulated genes by transcript profiling studies with tiling arrays using RNA from *sigW*, *prsW* or *rasP* mutant strains. Notably, these array analyses were performed in the absence of stress stimuli because, under these conditions σ^W^ exhibits a basal well-detectable level of activity, while stress-related side effects on the entire regulatory network are mostly absent. The absence of stress thus provides a unique opportunity to obtain an untroubled view of the σ^W^ regulon, even though σ^W^-regulated genes expressed at very low level might be missed. The results thus obtained were enriched using data from the *B. subtilis* transcript profiling study with tiling arrays in which gene expression in the parental strain 168 was assessed under 104 different biological conditions [35].

## Materials and Methods

### Bacterial strains, plasmids and growth conditions

The bacterial strains and plasmids used in this study are listed in Table 1. Strains were grown in Luria Bertani (LB) medium (Difco Laboratories) at 37°C with vigorous shaking. Overnight grown pre-cultures in LB medium were diluted to an OD_600_ of 0.05 in fresh LB medium and then grown to the exponential phase as determined by optical density readings. Under these conditions σ^W^ is active but the cells are not stressed.

**Table 1:** *B. subtilis* strains.

Strain	Genotype	Reference
168	*trpC2*	[35]
*sigW*	*trpC2 sigW::bleo*, Bm^r^	[42]
*rasP*	*trpC2 rasP::tc*, Tet^r^	[42]
*prsW*	*trpC2 prsW::bleo*, Bm^r^	[21]

### RNA isolation

Samples for three biological replicates of each mutant and the parental strain 168 were produced by independent culturing, harvesting of the bacterial cells, and RNA isolation. When the cultures reached an OD_600_ of 1.0 the equivalent of 15 OD units of cells were harvested and total RNA was isolated according to Eymann et al., 2002 [16] with some minor modifications. Cell culture samples were added to 0.5 volume of frozen killing buffer (20 mM Tris-HCl [pH 7.5], 5 mM MgCl_2_, 20 mM NaN_3_) and centrifuged for 10 min at 4°C. The cell pellets thus obtained were frozen in liquid nitrogen and stored at −80°C. Pellets were resuspended in 200 µl ice-cold killing buffer and transferred to precooled Teflon disruption vessels filled with liquid nitrogen. Cells were then disrupted for 2 min at 2600 rpm in a Mikro-Dismembrator S (Sartorius). The frozen powder was resuspended in 4 mL prewarmed (50°C) lysis solution (4 M guanidine thiocyanate, 25 mM sodium acetate [pH 5.2], 0.5% *N*-laurylsarcosinate [wt/vol]) and immediately frozen in liquid nitrogen. Total RNA was isolated by acid-phenol extraction. Samples were extracted twice with 1 volume of acid phenol/chloroform/isoamyl alcohol (25∶24∶1, [pH 4.5]) and once with 1 volume of chloroform/isoamyl alcohol (24∶1). After adding 1/10 volume of 3 M sodium acetate (pH 5.2), RNA was precipitated overnight with isopropanol at −20°C. Precipitated RNA was washed with 70% ethanol and dissolved in 100 µl of RNase free water. The isolated RNA was DNase-treated using the RNase-Free DNase Set (Qiagen) and purified using the RNA Clean-Up and Concentration Micro Kit (Norgen). RNA concentrations were measured using a Nanodrop-1000 spectrophotometer and RNA quality was assessed with the Agilent 2100 Bioanalyzer according to the manufacturer's instructions. Labeling of the samples and hybridizations were performed in strand-specific conditions by NimbleGen, as previously described [40], using Basysbio_T2 tiling arrays (NimbleGen). All tiling array data can be queried under the NCBI-GEO accession numbers GSE35236 and GPL15150.

### Statistical analyses

An aggregated expression measure was computed for each annotated and for each transcribed segment recently identified in the systematic study of transcriptome changes across lifestyles [35]. This measure consists of the median of the smoothed signal for probes with a unique perfect match on the genome sequence lying entirely within the boundaries of a particular feature [35]. The data was quantile-normalized to remove trends caused by technical variations between experiments [5]. A single linear model was fitted on the log2-scale data to assess the links between variations of expression and the genetic background of the analyzed *sigW*, *rasP* or *prsW* mutant strains and the parental strain 168. The p-values associated with the tests for non-null effects of each mutation compared to the parental strain were computed (function “lm” in R). One of the three hybridizations for the *prsW* mutant harbored an atypical transcriptome profile resembling that of RNA extracted from stationary phase cells. We interpret this observation as the result of a technical error when the samples were prepared, and this data point was therefore discarded. From the p-values, q-values allowing the control of the false discovery rate were estimated using the procedure of Strimmer [3] as implemented in the R package “fdrtool”. To increase the statistical power of our analyses, we also considered computation of false discovery rates using the same procedure, but restricting our attention to the subset of genes that were previously predicted as part of the global ECF regulon [35].

### Expression profiles across 104 conditions and ECF sigma factor binding site predictions

In addition to our transcript profiling experiments with mutant strains, we used the data from a study on the *B. subtilis* 168 transcriptome across 104 biological conditions (269 hybridizations), that was aimed at covering the maximum diversity of this bacterium's lifestyles [35]. These included growth on various media and carbon-sources, responses to stresses and developmental processes, such as competence development and the sporulation-germination cycle. In particular, we incorporated in our analysis the newly identified transcription segments, such as antisense RNAs and putative regulatory ncRNAs. For a high-level comparison of expression profiles, we relied on a classification based on average-linkage hierarchical clustering of the matrix of pairwise correlation with a cut-off set to 0.4 that defined 167 high-level clusters numbered in an arbitrary order C1 to C167. To complement the list of genes previously reported as being controlled by an ECF sigma factor, we also used the results of an un-supervised classification of the sequences upstream transcription start sites that identified 79 putative ECF sigma-factor dependent promoters [35].

## Results

### Two groups of down-regulated genes in *sigW* mutant cells

**Figure 1:**
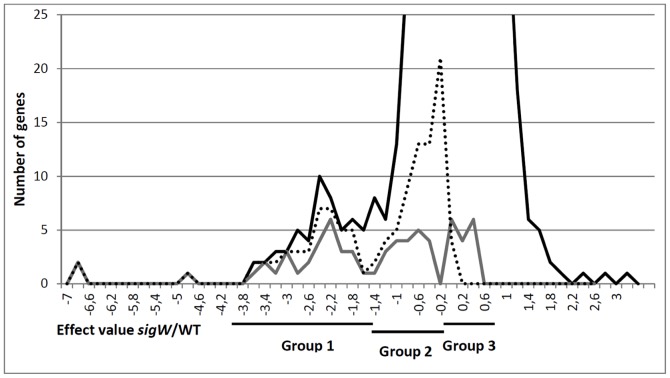
Effect values for transcriptional changes in *sigW* mutant *B. subtilis* cells. The transcript abundance in *sigW* mutant cells was compared to that in the parental strain 168 by tiling array analyses. The effect values were calculated on a log2 scale and the numbers of genes with a particular effect value were plotted as a function of the effect values. The black line represents all analyzed genes. The dashed line represents only the genes that are statistically significantly downregulated in the *sigW* mutant. The grey line represents the genes that were previously reported as being σ^W^-regulated. The groups 1, 2 and 3 of σ^W^-regulated genes are indicated.

Several previously documented studies have employed different strategies to identify genes that are regulated by σ^W^ [2], [6], [8], [9], [25], [26], [34], [46]. To accurately define the σ^W^ regulon and to include possible ncRNAs that are controlled by σ^W^ under non-stress conditions, we analyzed the genes that are down-regulated in the *sigW* mutant compared to the parental strain with tiling arrays (GEO accession number GSE35236). To ensure that genes not related to σ^W^ activity were excluded from this study, we made use of the fact that σ^W^ becomes active in the late exponential growth phase under non-stress conditions [25]. This is important because the absence of a stress stimulus provides a unique opportunity to obtain an untroubled view of the σ^W^ regulon since stress-related side effects on the entire regulatory network are largely absent. As expected, most genes previously designated as part of the σ^W^-regulon were down-regulated in our tiling array analysis in the *sigW* mutant compared to the parental strain. However, we observed that the effect amplitudes varied considerably between these genes, which allowed us to distinguish three subgroups (Figure 1, Tables 2 and 3). Group 1 consists of genes that are strongly down-regulated (this group has effect values ranging in log2-scale from −4 to −1.5). The most strongly down-regulated genes belonging to group 1 are *rsiW* and *spo0M*. Group 2 contains previously reported σ^W^-regulated genes that are less strongly down-regulated due to the *sigW* mutation than the genes in group 1 (effect-values between −1.5 and −0.2). Group 3 consists of 16 genes that were previously reported as σ^W^-regulated, but that nonetheless were not down-regulated in the present transcriptome analyses of the *sigW* mutant. Based on the present data, we identified 89 potentially σ^W^-regulated genes, which are located in 28 operons (Tables 2 and 3). The division of genes into groups 1 and 2 did not correlate with the transcription levels of these genes in the parental strain (Mann-Withney U-test p-value = 0.23). This rules out the possibility that the observed bimodal pattern of down-regulation of genes in the *sigW* mutant is simply a reflection of their transcription levels in the parental strain. Indeed, the apparently bimodal down-regulation pattern of gene expression in the *sigW* mutant probably results from more complex transcriptional regulation. Of the 28 identified σ^W^-regulated operons, 12 consist only of group 1 genes, and 8 consist only of group 2 genes. In 8 operons a combination of group 1 and group 2 genes was found, the group 2 genes always being localized at the end of these operons. In many cases, the boundary between group 1 and 2 genes correlated with the presence of an internal promoter (before *yozO*, *ybfP*, S161, *yxjH*, *ydjO*, S659, S716), or a terminator (after *ybfO*, *yvlD*, *ywrE*, *yqfB*) that could potentially be responsible for differences in their responses to the *sigW* deletion [35]. We also examined the sequences corresponding to predicted ECF Sigma factor binding sites [35] upstream of the genes of group 2 to those of group 1, but could not identify differences in the sequences that would explain the observed behavior.

**Table 2:** Down-regulated genes in *sigW* mutant cells.

Name	Effect sigW/WT	Function	Regulators	Genetic organization	Cluster	σ^WXY^ promoter sequence	Conclusion
*rsiW*	−6.84*	Control of *sigW* activity	σ^W^, AbrB	*sigW*-*rsiW*	C9	Yes	core σ^W^
*sigW*	−6.83*	Sigma W factor	σ^W^, AbrB	*sigW*-*rsiW*	C9	Yes	core σ^W^
*spo0M*	−4.92*	Sporulation	σ^W^, σ^H^	*spo0M*	C9	Yes	core σ^W^
S691	−3.66*			S691-*yoaG*-S690	C9	Yes	core σ^W^
*yeaA*	−3.61*		σ^W^, σ^E^	*yeaA-ydjP-ydjO*	C2	Yes	Secondary σ^W^
*ysdB*	−3.54*		σ^W^, σ^B^	*ysdB*	C9	Yes	core σ^W^
*yjoB*	−3.40*		σ^W^	*yjoB*	C9	Yes	core σ^W^
*ydjP*	−3.34*		σ^W^, σ^E^	*yeaA-ydjP-ydjO*	C2	Yes	Secondary σ^W^
S462 (indep)	−3.23*				C9	Yes	core σ^W^
*yxjI*	−3.12*		σ^W^, σ^E^, DegU	S1495-*yxjJ*-*yxjI*	C9	Yes	core σ^W^
*yoaG*	−3.07*		σ^W^	S691-*yoaG*-S690	C9	Yes	core σ^W^
*fosB*	−3.03*	Fosfomycin resistance	σ^W^	*fosB*-S658-S659	C9	Yes	core σ^W^
*ythP*	−2.98*	ABC transporter (ATP binding protein)	σ^W^	*ythP-ythQ*	C9	Yes	core σ^W^
S690	−2.90*			S691-*yoaG*-S690	C9	Yes	core σ^W^
S1495 (indep)	−2.89*			S1495-*yxjJ-yjxI*	C9	Yes	core σ^W^
*ythQ*	−2.74*	ABC transporter	σ^W^	*ythP-ythQ*	C9	Yes	core σ^W^
*S742*	−2.70*			S742-*yozO*-S740-S739-*yocM*	C9	Yes	core σ^W^
*pspA*	−2.68*		σ^W^, AbrB	*pspA-ydjG-ydjH-ydjI*	C6	Yes	Secondary σ^W^
*yfhL*	−2.52*	SdpC resistance	σ^W^, σ^B^	*yfhL-yfhM*	C5	Yes	Secondary σ^W^
*ydjG*	−2.51*		σ^W^, AbrB	*pspA-ydjG-ydjH-ydjI*	C6	Yes	Secondary σ^W^
S719 (inter)	−2.49*			*yobJ*-S719	C9	Yes	core σ^W^
S658 (inter)	−2.48*			*fosB*-S658-S659	C9	Yes	core σ^W^
*ybfO*	−2.47*		σ^W^, AbrB	*ybfO-ybfP-S89*	C9	Yes	core σ^W^
*ydbT*	−2.47*		σ^W^	*ydbS*-*ydbT*-S160-S162-*acpS*	C6	Yes	Secondary σ^W^
*ydbS*	−2.46*		σ^W^	*ydbS*-*ydbT*-S160-S162-*acpS*	C6	Yes	Secondary σ^W^
*pbpE*	−2.33*		σ^W^	*pbpE -racX*	C9	Yes	core σ^W^
*yuaG* (*floT*)	−2.33*	Sporulation (early stage)	σ^W^	*yuaF-yuaG-yuaI*	C9	yes	core σ^W^
*yfhM*	−2.30*	Survival of ethanol stress	σ^W^, σ^B^	*yfhL-yfhM*	C5	Yes	Secondary σ^W^
*ydjH*	−2.27*		σ^W^, AbrB	*pspA-ydjG-ydjH-ydjI*	C6	Yes	Secondary σ^W^
*yqfB*	−2.25	Resistance against sublancin	σ^W^	*yqeZ-yqfA-yqfB-yqfC-yqfD*	C9	Yes	core σ^W^
*yobJ*	−2.24*		σ^W^	*yobJ*-S719	C9	Yes	core σ^W^
*yqeZ*	−2.21	Serine protease, Resistance against sublancin	σ^W^	*yqeZ-yqfA-yqfB-yqfC-yqfD*	C9	Yes	core σ^W^
*ydjI*	−2.17*		σ^W^, AbrB	*pspA-ydjG-ydjH-ydjI*	C6	Yes	Secondary σ^W^
*racX*	−2.12*	Control of biofilm formation	σ^W^	*pbpE -racX*	C9	Yes	core σ^W^
*yqfA*	−2.11	Resistance against sublancin	σ^W^	*yqeZ-yqfA-yqfB-yqfC-yqfD*	C9	Yes	core σ^W^
*mtlF*	−2.05	Uptake of mannitol	MtlR	*mtlA-mtlF-mtlD*	C36	No	background
*yuaI*	−2.02*		σ^W^	*yuaF-yuaG-yuaI*	C9	Yes	core σ^W^
*mtlD*	−1.97	Mannitol utilization	MtlR	*mtlA-mtlF-mtlD*	C36	No	background
*yvlA*	−1.91*		σ^W^, AbrB	*yvlA-yvlB-yvlC-yvlD*-S1338	C9	Yes	core σ^W^
*yvlB*	−1.85*		σ^W^, AbrB	*yvlA-yvlB-yvlC-yvlD*-S1338	C9	Yes	core σ^W^
*mtlA*	−1.85	Mannitol utilization	MtlR	*mtlA-mtlF-mtlD*	C36	No	background
*ywrE*	−1.82		σ^W^	*ywrE*-S1390	C9	Yes	core σ^W^
*yuaF*	−1.78*		σ^W^	*yuaF-yuaG-yuaI*	C9	Yes	core σ^W^
*yoaF*	−1.58		σ^W^	*yoaF*	C48	Yes	Secondary σ^W^
S160 (inter)	−1.56			*ydbS*-*ydbT*-S160-S162-*acpS*	C6	Yes	Secondary σ^W^
*ybfP*	−1.38*		σ^W^, AbrB	*ybfO-ybfP-S89*	C9	Yes	core σ^W^
S89*	−1.34*			*ybfO-ybfP-*S89	C9	Yes	core σ^W^
*yvlD*	−1.34		σ^W^,AbrB	*yvlA-yvlB-yvlC-yvlD-*S1338	C9	Yes	core σ^W^
*yvlC*	−1.32		σ^W^,AbrB	*yvlA-yvlB-yvlC-yvlD-*S1338	C9	Yes	core σ^W^
*yjzH*	−1.19			*yjzH*-S442	C9	Yes	core σ^W^
*sppA*	−1.18	Signal peptide peptidase	σ^W^	*sppA-yteJ*	C6	Yes	Secondary σ^W^
*yteJ*	−1.17*		σ^W^	*sppA*-*yteJ*	C6	Yes	Secondary σ^W^
*yaaN*	−1.11		σ^W^	*xpaC*-*yaaN*-S22	C9	Yes	core σ^W^
*yceE*	−1.04	Resistance against ethanol stress and cold	σ^W^, σ^B^, σ^M^	S106-*yceC*-*yceD*-*yceE*-*yceF*-*yceG*-*yceH*	C6	Yes	Secondary σ^W^
*S716*	−0.95			Downstream of *yobJ*-S719	C31	Yes	Read through
S659 (indep)	−0.94			*fosB*-S658-S659	C9	Yes	core σ^W^
*yceD*	−0.90	Resistance against ethanol stress	σ^W^, σ^B^, σ^M^	S106-*yceC*-*yceD*-*yceE*-*yceF*-*yceG*-*yceH*	C6	Yes	Secondary σ^W^
*yceH*	−0.88*		σ^W^, σ^B^, σ^M^	S106-*yceC*-*yceD*-*yceE*-*yceF*-*yceG*-*yceH*	C6	Yes	Secondary σ^W^
S22 (intra)	−0.88			*xpaC*-*yaaN*-S22	C9	Yes	core σ^W^
*yceG*	−0.87*		σ^W^, σ^B^, σ^M^	S106-*yceC*-*yceD*-*yceE*-*yceF*-*yceG*-*yceH*	C6	Yes	Secondary σ^W^
*yceC*	−0.84		σ^W^, σ^B^, σ^M^	S106-*yceC*-*yceD*-*yceE*-*yceF*-*yceG*-*yceH*	C6	Yes	Secondary σ^W^
*yxjH*	−0.83		S-box	Downstream of S1495-*yxjJ*-*yxjI*	C48	Yes	Read through
*ygzA*	−0.82			Opposite of *spo0M*	C2	No	Background
S1338	−0.80			*yvlA-yvlB-yvlC-yvlD-*S1338	C9	Yes	core σ^W^
*ilvD*	−0.78	Aminoacid biosynthesis	CodY		C48	No	background
*yknX*	−0.78	Resistance against SdpC	σ^W^,AbrB	*yknW-yknX-yknY-yknZ*	C9	Yes	core σ^W^
S106	−0.78			S106-*yceC*-*yceD*-*yceE*-*yceF*-*yceG*-*yceH*	C6	Yes	Secondary σ^W^
*xpaC*	−0.77		σ^W^	*xpaC*-*yaaN*-S22	C9	Yes	core σ^W^
*yqfC*	−0.76*		σ^E^	*yqeZ*-*yqfA*-*yqfB*-*yqfC*-*yqfD*	C2	Yes	Secondary σ^W^
*yknY*	−0.76	Resistance against SdpC	σ^W^,AbrB	*yknW-yknX-yknY-yknZ*	C9	Yes	core σ^W^
S1175	−0.75			5′ *mntA*	C1	No	background
*yceF*	−0.74		σ^W^, σ^B^, σ^M^	S106-*yceC*-*yceD*-*yceE*-*yceF*-*yceG*-*yceH*	C6	Yes	Secondary σ^W^
*yqfD*	−0.72*		σ^E^	*yqeZ*-*yqfA*-*yqfB*-*yqfC*-*yqfD*	C2	Yes	Secondary σ^W^
*yknZ*	−0.69	Resistance against SdpC	σ^W^,AbrB	*yknW-yknX-yknY-yknZ*	C9	Yes	core σ^W^
*mtnK*	−0.65		S-box	*mtnK-mtnA*	C48	No	background
*alsD*	−0.63			*alsS-alsD*	C39	No	background
*yozO*	−0.60		σ^W^	S742-*yozO*-S740-S739-*yocM*	C9	Yes	core σ^W^
*yknW*	−0.57	Resistance against SdpC	σ^W^,AbrB	*yknW-yknX-yknY-yknZ*	C9	Yes	core σ^W^
S740 (inter)	−0.54			S742-*yozO*-S740-S739-*yocM*	C6	Yes	Secondary σ^W^
S161	−0.52	Fatty acid biosynthesis		5′ *acpS*	C3	Yes	Secondary σ^W^
S739	−0.51			S742-*yozO*-S740-S739-*yocM*	C2	Yes	Secondary σ^W^
S1390 (inter)	−0.48			*ywrE*-S1390	C9	Yes	core σ^W^
S442 (inter)	−0.48			*yjzH*-S442	C9	Yes	core σ^W^
*acpS*	−0.45	Fatty acid biosynthesis		*ydbS*-*ydbT*-S160-S162-*acpS*	C3	Yes	Secondary σ^W^
S162	−0.44			S162-*ydcC*	C2	Yes	Secondary σ^W^
*ydcC*	−0.42		σ^E^	S162-*ydcC*	C2	Yes	Secondary σ^W^
*thiC*	−0.41	Thiamine biosynthesis	Thi-box	Downstream of *ygzA*	C48	No	Background
*ydjO*	−0.41		σ^W^, σ^E^	*yeaA-ydjP-ydjO*	C2	Yes	Secondary σ^W^
*yocM*	−0.41			S742-*yozO*-S740-S739-*yocM*	C2	Yes	Secondary σ^W^

Only the down-regulated genes with effect values lower than −0.4 and p-values lower than 0.05 are shown. Effect values marked with * have q-values of less than 0.05. For each individual gene, the Table lists the function, the previously identified regulation, the genetic organization, the condition-dependent transcription profile cluster as defined by Nicolas *et al* [35], the presence of a predicted ‘σ^WXY^’ promoter sequence [35], and our conclusion whether it belongs to the σ^W^ core regulon or the secondary σ^W^-regulated genes. It should be noted here that the previously predicted ‘σ^WXY^’ promoter sequence [35] also covers the potential binding site for σ^M^. The division between group 1 and group 2 genes is indicated by a bold line.

**Table 3:** Previously Reported σ^W^-regulated genes that were not significantly down-regulated in the *sigW* mutant strain.

Name	Effect sigW/WT	Function	Regulation	Genetic organization	Cluster	σ^WXY^ promoter sequence	Conclusion
*yxzE*	−0.45		σ^W^,AbrB	*yxzE*-S1489	C9	Yes	core σ^W^
S1489	−0.31			*yxzE*-S1489	C9	Yes	core σ^W^
*bscR (fatR)*	−0.15	Fatty acid biosynthesis	σ^M^, σ^W^, σ^X^, FatR	*yrhH-bscR-yrhJ*	C10	Yes	σ^M^
*ywbO*	−0.02	Iron uptake	σ^M^, σ^W^, σ^X^, Fur	*ywbN-ywbO*	C29	No	Not σ^W^ - Fur-regulated
*fabHa*	−0.03	Fatty acid biosynthesis	σ^W^, FapR	*fabHA-fabF*	C3	Yes (P5 +114)	σ^W^ Kingston et al 2011
*efeN (ywbN)*	−0.04		σ^M^, σ^W^, σ^X^, Fur	*ywbL-ywbM-ywbN* *ywbN-ywbO*	C29	No	Not σ^W^ - Fur-regulated
*fabF*	−0.07	Fatty acid biosynthesis	σ^W^, FapR	*fabHA-fabF*	C3	Yes	σ^W^ Kingston et al 2011
*yrhJ (cypB)*	−0.08		σ^M^, σ^W^, σ^X^	*yrhH-fatR-yrhJ*	C10	Yes	σ^M^
*ywaC*	0.06	(p)ppGpp synthetase	σ^M^, σ^W^		C79	Yes	σ^M^
*yjbC*	0.07		PerR, σ^B^, σ^M^, σ^W^, σ^X^	*yjbC-spx*	C5	Yes	σ^B^ and σ^M^
*yjbD (spxA)*	0.17		PerR, σ^B^, σ^M^, σ^W^, σ^X^	*yjbC-spx*	C17	Yes	σ^B^ and σ^M^
*divIC*	0.23	Septum formation	σ^E^, σ^M^, σ^W^, σ^X^	*yabM-yabN-yabO-yabP-yqabQ-divIC-yabR*	C20	Yes	σ^M^
*yrhH*	0.25		σ^M^, σ^W^, σ^X^	*yrhH-fatR-yrhJ*	C10	Yes	σ^M^
*abh*	0.30	Gene regulation during transition phase	σ^W^, σ^X^		C36	Yes	σ^X^
*ywnJ*	0.31		σ^F^, σ^M^, σ^W^, σ^X^		C2	Yes	σ^M^
*bcrC*	0.33	Resistance to bacitracin and oxidative stress	σ^I^, σ^M^, σ^W^, σ^X^		C10	Yes	σ^M^
*yqjL*	0.37	Resistance against paraquat	σ^B^, σ^M^, σ^W^		C78	Yes	σ^B^, σ^M^

For each individual gene, the Table lists the function, the previously identified regulation, the genetic organization, the condition-dependent transcription profile cluster as defined by Nicolas *et al* [35], the presence of a predicted ‘σ^WXY^’ promoter sequence [35], and our conclusion whether it belongs to the σ^W^ core regulon or the secondary σ^W^-regulated genes. It should be noted here that the previously predicted ‘σ^WXY^’ promoter sequence [35] also covers the potential binding site for σ^M^.

Genes that were found to be statistically significantly down-regulated in the *sigW* mutant are likely to be regulated by σ^W^. To establish this list of genes we computed q-values from the p-values, which allowed us to control the number of false positive identifications by taking into account the high number of genes examined. Based on this statistical analysis, we propose that genes down-regulated in the *sigW* mutant with q-values lower than 0.05 are most likely genuine σ^W^-regulated genes (Table 2; genes with q-values<0.05 are marked with *). However, if we consider only these genes as being σ^W^-regulated, several genes that were previously shown to be σ^W^-regulated by other methods (Table S1) would have to be discarded from the σ^W^ regulon under non-stress conditions despite their apparent down-regulation. To avoid such potentially false negative exclusions, we maintained all the genes that were down-regulated with q-values higher than 0.05 but p-values lower than 0.05 also in our shortlist of potentially σ^W^-regulated genes. These genes were further analyzed by assessing their transcription profiles under 104 conditions, including several conditions known to induce SigW.

### Definition of the σ^W^ regulon by assessment of transcript profiles across conditions

**Figure 2:**
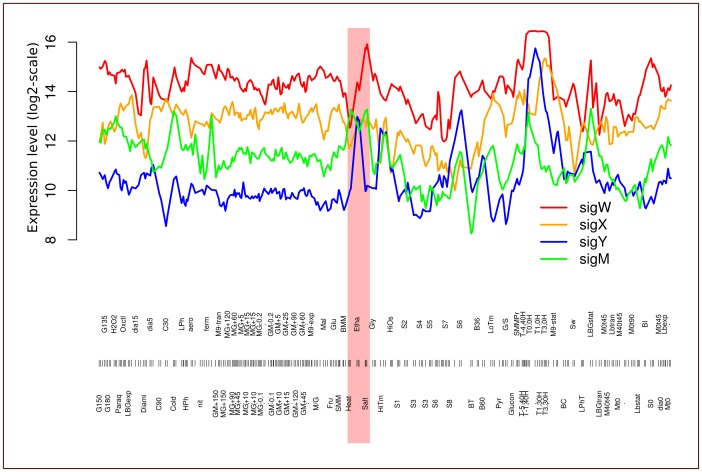
Expression profiles of *sigW*, *sigX*, *sigY* and *sigM* in *B. subtilis* 168 across 104 conditions. The 269 tiling array hybridizations [35] are arranged along the x-axis. Of particular interest for discriminating the activities of the encoded sigma factors are the conditions heat stress (‘heat’), ethanol stress (‘etha’) and hypersaline stress (‘salt’), which are marked by pink shading.

**Figure 3:**
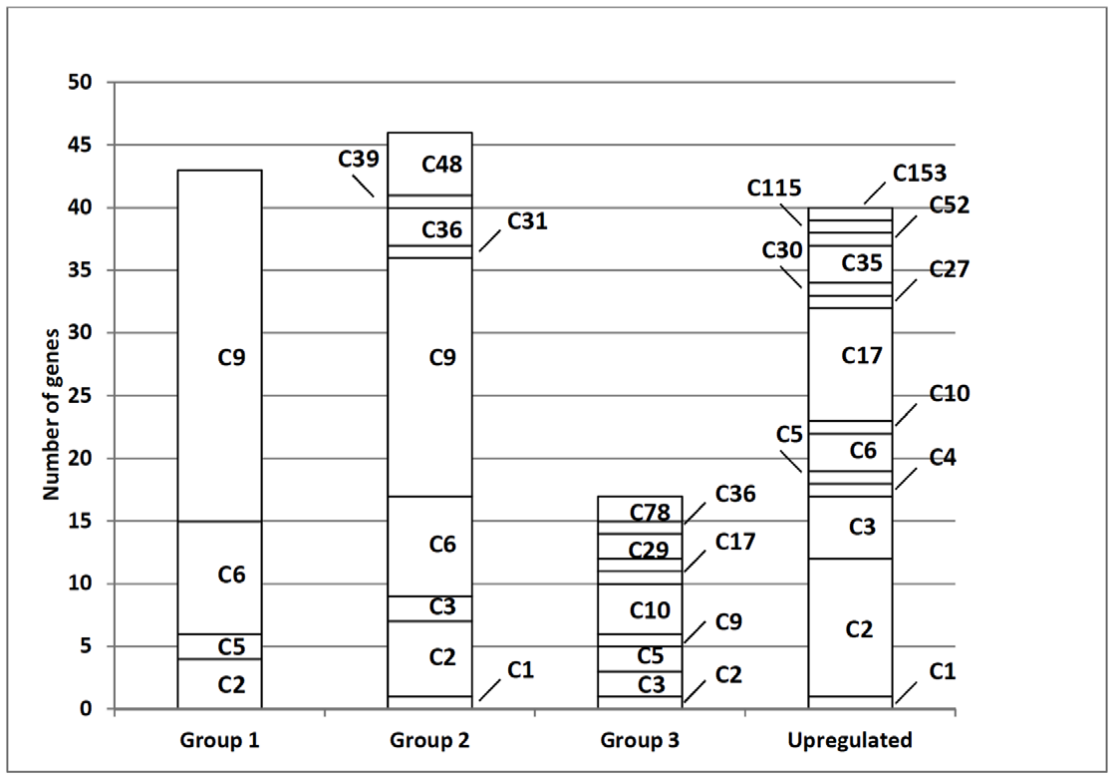
Assignment of clusters of genes with related transcript profiles across conditions to different groups of genes that are down-regulated or up-regulated in *sigW* mutant cells. The down-regulated genes are represented by groups 1 and 2 (see also Fig. 1). Genes in group 3 were previously reported as σ^W^-regulated, but our present studies provided no evidence for their proposed σ^W^-dependency (see Fig. 1). The up-regulated genes are represented in a separate bar. Previously defined transcription clusters [35] are indicated in each bar by their C-number.

**Figure 4:**
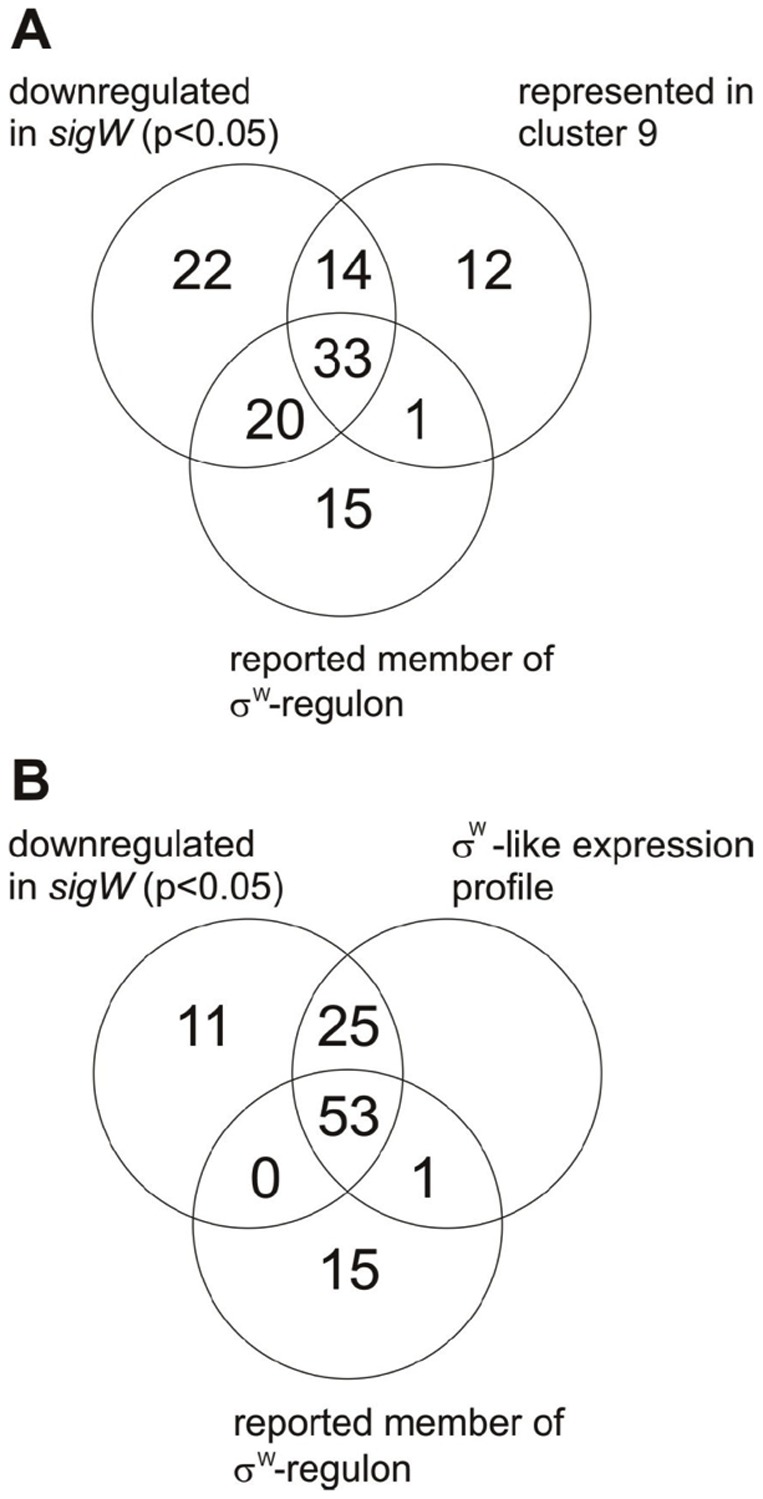
Venn diagrams for the comparison of genes that were found to be downregulated in the *sigW* mutant strain with previously reported σ^W^-regulated genes and genes that display similar condition-dependent transcription profiles as *sigW*. Diagram A includes only the so-called cluster C9 genes that have highly similar condition-dependent transcription profiles as defined by Nicolas *et al* [35]. Notably, the *sigW* gene is included in cluster C9. Diagram B includes all genes that show condition-dependent expression profiles similar to that of *sigW*, including induction upon salt stress.

To minimize the false positive identifications of σ^W^-regulated genes, we took advantage of a large-scale tiling array analysis of gene expression in *B. subtilis* 168 across 104 conditions, involving 269 hybridizations [35]; GEO accession number GPL15150). Within this previous study promoters of different sigma factors were classified based on an unsupervised algorithm. Notably, σ^W^ regulated promoters were classified together with the other ECF sigma factors (σ^W^, σ^X^, σ^Y^ and σ^M^) as having sigma factor binding sites of the ‘σ^WXY^’ type, because no distinction between promoters recognized by sigma factors with similar DNA binding motifs could be made (note that although this binding site was annotated as ‘σ^WXY^’ type, it also covers the σ^M^ binding site). Importantly, the results of this study revealed marked differences in the transcription profiles of the *sigW*, *sigY*, *sigX* and *sigM* genes across conditions, especially during heat, salt and ethanol stress (Figure 2). This was an important observation, because it can help in the dissection of the respective regulons. The analysis of transcription profiles across the 104 conditions showed that the transcription profiles of 59 genes cluster with that of *sigW* in the previously defined transcription cluster C9 (Figure 3, [35]). Importantly, most genes in cluster C9 were found to be significantly down-regulated in the *sigW* mutant in our present studies and/or were previously reported as σ^W^-regulated (Figure 4). The 12 genes within cluster C9 that are not σ^W^-regulated represent members of the σ^Y^ regulon, including the *sigY* gene itself. Their presence in cluster C9 relates to the fact that σ^Y^-regulated genes behave quite similarly to σ^W^-regulated genes, the main distinguishing feature being that they are induced by ethanol stress rather than salt stress. Clearly, the known σ^Y^-regulated genes in cluster C9 were not down-regulated in the *sigW* mutant, whereas all other genes in cluster C9 were down-regulated in the *sigW* mutant (Figure 4A). Only one gene in cluster C9, *yxzE*, which was previously reported to be σ^W^-regulated, did not qualify as a σ^W^-regulated gene in our statistical analyses as its down-regulation in the *sigW* mutant (effect value −0.45) had a p-value of 0.08. However, based on the combined data, we believe that *yxzE* should be regarded as a member of the σ^W^ regulon. Accordingly, the long 3′ UTR of *yxzE* with the designation S1489 is probably also part of the σ^W^ regulon, which is supported by the fact that it is present in cluster C9 (Table 3).

20 genes that have previously been reported as σ^W^-regulated were also found to be down-regulated in the *sigW* mutant, but are nevertheless not included in cluster C9 (Figure 4A). 14 of these genes belong to cluster C6 (Figure 3), whereas the others are distributed over several other clusters. Possibly, these genes are not only regulated by σ^W^, but also by other sigma factors or gene regulators, which would lead to expression profiles that differ from the *sigW* expression profile. Therefore, we examined the expression profiles of these genes with special attention to induction during salt stress, which is a hallmark of the σ^W^-regulated genes [18], [38], [43]. In addition, we also compared these profiles with the profiles of genes in the σ^M^, σ^X^ and σ^Y^ regulons that also respond to cell envelope stress. These analyses revealed in total 79 genes with ‘σ^W^-like’ expression profiles that are induced upon salt stress (i.e. 54 previously reported members of the σ^W^ regulon *plus* 25 newly identified σ^W^-regulated genes; Figure 4B). Based on the transcriptional profiles under different conditions, and the requirement to be down-regulated in the *sigW* mutant, we propose to make a distinction between core genes of the σ^W^-regulon and secondary σ^W^-regulated genes. The σ^W^-regulated genes in cluster C9 would be the core genes of the σ^W^ regulon and all other σ^W^-dependent genes would be secondary σ^W^ regulon genes (Table 2).

The genes that were newly identified as being σ^W^-regulated were mainly novel ncRNAs that are part of σ^W^-regulated operons (Table 2). One distinct exception is the ncRNA S462, which is located downstream of *htrA*. S462 is an independent ncRNA that is preceded by a consensus σ^WXY^ binding site [35]. *yjzH* and the downstream ncRNA S442 also represent novel members of the σ^W^ regulon, which are preceded by a predicted σ^WXY^ binding sequence. Additionally, in several occasions there was read-through from σ^W^-regulated operons into downstream genes. For example, the operon *yqeZ*-*yqfA-yqfB* is known to be σ^W^-regulated, but the downstream genes *yqfC* and *yqfD* had previously not been identified as being σ^W^-regulated. Although *yqfC* and *yqfD* were not as strongly down-regulated in the *sigW* mutant as the preceding operon, the down-regulation of these genes was still clearly significant with q-values of less than 0.05. Additionally, these genes were found to be up-regulated during salt stress [35]. Therefore, we conclude that *yqfC* and *yqfD* are truly σ^W^-regulated. In other cases of read through no induction during salt stress was observed, and the respective genes are therefore not considered to be σ^W^-regulated.

**Figure 5:**
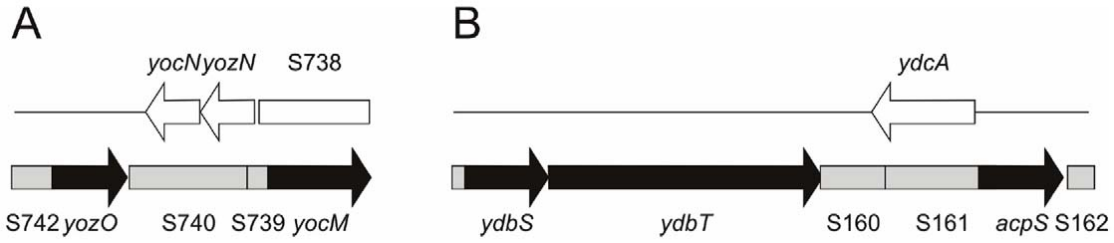
Organization of complex σ^W^-regulated operons. The σ^W^-regulated ORFs are indicated in black, and the σ^W^-regulated ncRNAs are indicated in grey. Genes and an ncRNA on the opposite strand are indicated in white. A, The *yozO*-*yocM* operon. B, The *ydbST*-*acpS* operon.

Several genes further downstream of known σ^W^-regulated operons also behave like σ^W^-regulated genes. Downstream of *yozO* for example, S740, S739 and *yocM* are all down-regulated in the *sigW* mutant and induced upon salt stress (Figure 5A). Similarly, downstream of the *ydbST* operon, S161, *acpS* and S162 are down-regulated in the *sigW* mutant and induced upon salt stress (Figure 5B). In other cases the situation is different. For example, *ygzA*, a gene starting close to the start site of *spo0M*, but running in the opposite direction, is also down-regulated in the *sigW* mutant. Nevertheless, *ygzA* is not preceded by a consensus binding sequence for σ^WXY^, and this gene is also not induced by salt stress. Likewise, the *yxjH* gene downstream of the σ^W^-regulated gene *yxjI* is down-regulated in the *sigW* mutant, but also in this case no induction is observed during salt stress. Thus, we do not consider *ygzA* and *yxjH* to be genuinely σ^W^-regulated genes.

15 genes that were previously reported to be σ^W^-regulated were not down-regulated in the *sigW* mutant (Table 3, Figures 1, 3 and 4). This observation cannot be explained by a simple absence of expression of these genes in the parental strain that would have precluded the possibility to observe their down-regulation. This view is supported by the finding that the distribution of the expression levels of these genes in the parental strain was not significantly different from the distribution of the expression levels of genes belonging to groups 1 and 2 (Mann-Withney U-test p-value of 0.64). Indeed, these genes have been assigned to multiple σ regulons besides the σ^W^ regulon and they mostly appear to show condition-dependent transcription profiles that are more similar to those of genes regulated by σ factors other than σ^W^ (Table 3). We therefore examined whether these genes had been previously shown to act in a typical σ^W^-dependent manner, or whether their dependency on other ECF sigma factors had been shown (Table S1). The majority of these 15 genes do not show a typical upregulation pattern under conditions inducing the σ^W^ regulon. 11 of the 15 genes have been shown to be regulated by other sigma factors (10 by σ^M^ and 1 by σ^X^). *ywnJ*, *ywbN* and *yrhH* have only been shown to have the potential for binding σ^W^ *in vitro* [7], [8], [25], and no *in vivo* data suggest a σ^W^-dependence of their promoters. *fabHa* has been shown to be expressed σ^W^-dependently [31], and upregulation of the *fabHa-fabF* operon has been reported upon overexpression of σ^W^ [2]. However, this operon was never observed to be upregulated in any of the conditions known to induce the σ^W^ regulon. This is somewhat surprising, but may be explained by the promoter being located within the *fabHa* gene itself. The majority of these 15 genes are therefore unlikely to be σ^W^-regulated.

Lastly, 40 genes appeared to be up-regulated in the *sigW* mutant with effect values of more than 0.4 and p-values of less than 0.05 (Table 4). However, it should be noted that none of these changes have q-values smaller than 0.05. This suggests that these up-regulations may represent false positive results or indirect effects that are not as strong as direct regulatory effects. Several of the up-regulated genes are located in the close proximity of σ^W^-regulated genes, but are encoded by the opposite strand. Two of these genes, *ybbK* and *ybbJ*, are located immediately opposite of *sigW* and, therefore, the up-regulation of these genes in the *sigW* mutant could be the result of a polar effect of the deletion of *sigW*. However, the transcription profiles of both of these genes do not show changes during exposure to high salt and the same is true for the other up-regulated genes. Therefore, we do not consider *ybbK*, *ybbJ* and other genes up-regulated in the *sigW* mutant as novel σ^W^-regulated genes.

**Table 4:** Genes that were up-regulated in the *sigW* mutant.

Name	Effect sigW/WT	Function	Regulation	Genetic organization	Cluster
*ybbK*	3.07			*ybbK-ybbJ* Opposite of *sigW* (↓)	C6
*ybbJ*	2.68			*ybbK-ybbJ* Opposite of *sigW* (↓)	C6
S928 (inter)	2.25			Between *mgsR* and *rsbRD*	C5
S1380	1.16				C10
*ykzV*	1.13				C2
S1026 (inter)	0.92			Upstream of *yrzI* (↑)	C2
*cotT*	0.91				C2
*yodI*	0.83		σ^K^		C2
S1030	0.82			5′ of *yrhF*,	C3
*murG*	0.77	Peptidoglycan precursor biosynthesis	σ^E^, σ^M^,SpoIID		C4
S981	0.73			3′ of *yqaP*, opposite of *yqaR* (↑) and *yqbC* (↑)	C17
*ymaG*	0.68	Spore coat protein	σ^K^		C2
S655	0.66			Opposite of *fosB* (↓)	C17
S862	0.65			5′ of *spoIVA*	C2
S1356	0.64			5′ of *degS* (↑)	C3
*yrzI*	0.63				C2
S613	0.62			5′ of *ymzD* (slightly ↑)	C27
S663	0.61			5′ of *ccdA* (slightly ↑)	C17
S1405 (inter)	0.60			Downstream of *spoIID* (slightly ↑)	C2
S254 (indep)	0.60				C17
*ykzW*	0.59	RNA that inhibits AhrC translation	CcpN regulon		C30
S653 (indep)	0.57			Downstream of *fosB* (↓)	C17
*ydeH*	0.56		AbrB		C17
*yqaR*	0.54			Close to S981 (↑)and *yqbC* (↑)	C6
S360 (inter)	0.54				C35
S118 (inter)	0.52			Opposite of *yuaI-yuaF-yuaG*	C52
*obg*	0.50	Ribosome assembly (essential), possibly required for Spo0A-activation			C3
*cotU*	0.50	Spore coat protein	GerE, GerR		C2
*yqxD*	0.46		σ^H^	upstream of S952 (slightly ↑)	C153
S278	0.46			5′ *yfzA* (↑)	C17
*pssA*	0.46	Biosynthesis of phospholipids		Upstream of *ybfO-ybfP*(↓)	C3
S303	0.45			5′ of *ygxA*	C3
*comK*	0.44	Competence and DNA uptake regulation	AbrB, ComK, DegU, CodY, Rok		C1
*yktD*	0.43				C115
S1543 (intra)	0.43			Upstream of *yydI, yydJ* (both slightly ↑)	C35
S95	0.42			5′ of *ycbJ*	C35
S831	0.42			5′ of *ypeP*	C2
S427	0.42			5′ of *yjzE*	C2
S924	0.41			5′ of *sinI*	C17
*yfzA*	0.41			S278(↑)-*yfzA*	C17

Only the genes with Effect values higher than 0.4 and p-values lower than 0.05 are shown. Arrows behind genes in the ‘genetic organization’ column indicate whether the transcription of these genes was up- (↑) or down-regulated (↓). For each individual gene, the Table lists the function, the previously identified regulation, the genetic organization, and the condition-dependent transcription profile cluster as defined by Nicolas *et al* [35].

### Function of the σ^W^ regulon

The σ^W^ regulon is responsible for activating genes whose products are likely to be needed upon envelope stress, or beneficial under conditions of alkali shock, salt stress and treatment with cationic peptides and agents that impair cell wall biosynthesis [9], [18], [38], [39], [43]. To verify this view, the genes identified in our study as being part of the σ^W^ regulon were analysed for function according to their classification in *Subti*Wiki [17] (Table 2, Table S1). Indeed, the groups of genes that were most largely represented encoded cell envelope stress proteins, membrane proteins and proteins involved in resistance against toxins or antibiotics. These proteins have been implicated in protecting the cell from stresses that affect the membrane and in detoxification upon contact with toxic compounds. Our present findings suggest that, also under non-stress conditions, it may be beneficial for *B. subtilis* to express the respective σ^W^-regulated genes at a basal level, for example to allow fast and effective responses to any membrane stresses that may suddenly occur. Notably, over half of the genes identified as being σ^W^-regulated are *B. subtilis* ‘y’ genes, essentially genes that have yet to be functionally annotated. Therefore, until the functions of these genes are defined it will remain difficult to determine which σ^W^-regulated genes function in what capacity when the regulon is upregulated.

### Comparison of global transcription in *rasP*, *prsW* and *sigW* mutant cells

Deletion of the genes for RasP and PrsW under stress conditions inhibits the activation of the σ^W^-regulon, because both of these proteases are required for inactivation of the σ^W^ anti-sigma factor RsiW. Thus, no activation of σ^W^-controlled genes was detectable in *rasP* or *prsW* mutant cells upon stress [15], [19], [21], [42]. In addition, the *rasP* mutant is known to display several phenotypes, such as defects in competence and protein secretion, which are not observed in *prsW* or *sigW* mutants [20], [32], [41], [47]. During membrane protein overproduction, the *rasP* mutant also behaves differently from the *prsW* and *sigW* mutants. Whereas *prsW* and *sigW* mutations generally improve membrane protein overproduction, in the *rasP* mutant overproduction of all tested membrane proteins was abolished [50].

**Figure 6:**
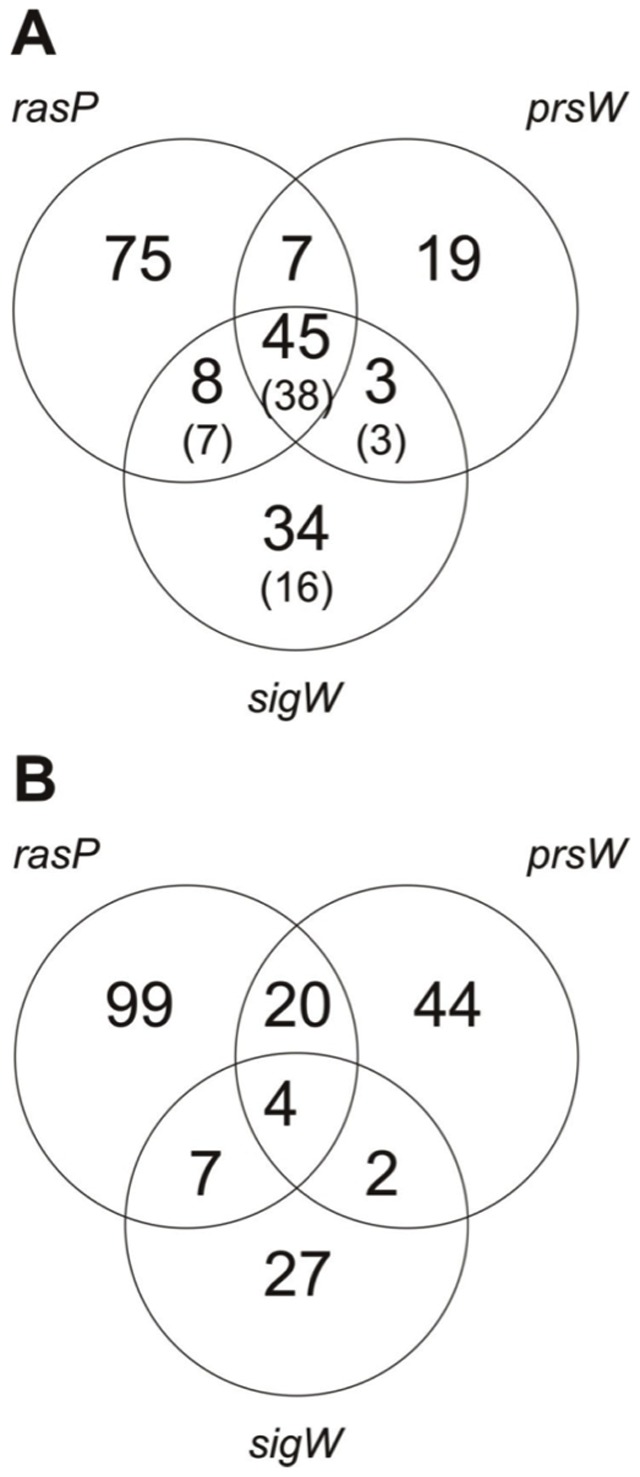
Up- and down-regulation of genes in *rasP*, *prsW* or *sigW* mutant strains compared to the wild-type. A, Venn diagram for down-regulated genes. B, Venn diagram for upregulated genes. Only genes with transcriptional changes that have p-values lower than 0.05 and effect values lower than −0.40 (A) or higher than 0.40 (B) are included. The genes that are considered to be σ^W^-regulated are indicated between brackets.

We wanted to know whether RasP and PrsW, the genes of which are both expressed under the tested non-stress conditions, play a role in the control of the basal activity of the σ^W^ regulon. Generally, the transcriptional changes in the *rasP* or *prsW* mutant strains compared to the parental strain and the *sigW* mutant were rather small and only few had q-values below 0.05 (15 in the *rasP*/WT comparison, 0 in the *prsW*/WT comparison, 21 in the *rasP*/*sigW* comparison, and 14 in the *prsW*/*sigW* comparison). Closer examination revealed that only 3 genes associated with q-values below 0.05 were not predicted to belong to the global ECF regulon defined in Nicolas *et al.* [35] (i.e. *natA*, *hisG* and *tetB*). We therefore reasoned that statistical power could be increased by searching for differential expression in priority among the 243 genes and new expression segments included in this analysis that were previously classified as members of the global ECF regulon [35]. Indeed, the estimates that we obtained for the false discovery rates of global ECF regulon genes with p-values≤0.05 were 9.7% for the *sigW*/WT comparison, 10.3% for the *rasP*/WT comparison, 12.3% for the *prsW*/WT comparison, 11.8% for the *rasP*/*sigW* comparison and 13.4% for the *prsW*/*sigW* comparison. These genes are listed in Tables S2, S3, S4. For completeness, other genes with p-values≤0.05 have also been listed although they probably include a much higher fraction of false discoveries. Altogether, the composition of these lists revealed that the afore-described σ^W^-regulated genes were down-regulated in both the *rasP* and *prsW* mutants, but to lesser extents than in the *sigW* mutant. This indicates that the deletion of *rasP* or *prsW* indeed decreased the activity of σ^W^, but that σ^W^ activity was not completely abolished in the respective mutants under the applied non-stress conditions (Figure 6, Table S2A). Apparently, some σ^W^ molecules were able to escape from binding to RsiW, even in the absence of RasP or PrsW, thereby causing low-level expression of the σ^W^ regulon that was independent from intramembrane proteolysis by RasP and PrsW. Among the non-σ^W^-regulated genes that were down-regulated in the *rasP* mutant were several genes that are involved in the development of genetic competence (i.e. *oppA*, *nucA*, *ssbB*, *rapD*). Other genes that were specifically down-regulated in the *rasP* mutant mainly relate to lipid and cell wall turnover.

In both the *rasP* and *prsW* mutant strains, slight increases in transcription were detected for genes involved in compatible solute transport, which is important for osmoregulation (Table S2). Even though not all of these genes were always significantly up-regulated in each mutant, there seemed to be a mild, general up-regulation of these genes in both the *rasP* and *prsW* mutant strains. Additionally, slightly increased transcription of genes involved in teichoic acid synthesis, phospholipid biosynthesis, cell wall biogenesis and cell shape was observed. Genes that were specifically up-regulated in the *rasP* mutant include genes involved in amino acid metabolism (e.g. genes for histidine and arginine biosynthesis, and ornithin and citrullin utilization) and genes involved in cell envelope stress systems (e.g. the *natAB-yccK* operon [11], [36], [37], the LiaRS, WalRK [4], [13] and DesRK two-component systems, and the σ^M^-regulon [14], [28], [34]). However, not all genes regulated by these systems were up-regulated and therefore the significance of these findings remains unclear.

Notably, in our previous studies we have reported significantly increased levels of HtrA and HtrB in the *rasP* mutant [50]. Nevertheless, the *cssR* and *cssS* transcription levels were only slightly down-regulated in the *rasP* mutant and the same was true for the *sigW* or *prsW* mutant strains (Table 5). Furthermore, the transcription of the CssRS-regulated *htrA* and *htrB* genes was not significantly altered in *rasP*, *prsW* or *sigW* mutant cells (Table 5). This implies that the activity of the CssRS system is not responsible for the increased HtrA and HtrB levels in the *rasP* mutant.

**Table 5:** Transcriptional changes of genes regulated by the CssRS two-component system.

	Effect sigW/WT	Effect rasP/WT	Effect prsW/WT
***cssR***	−0.20	−0.20	−0.19
***cssS***	−0.22	−0.22	−0.03
***htrA***	−0.24	0.21	0.07
***htrB***	−0.29	0.18	0.06

Lastly, a direct comparison of global transcription in the *rasP* and *sigW* mutant strains resulted in very few statistically significant changes (Tables S3 and S4). Compared to the *sigW* mutant, a few genes including *rocD* and *rocA*, *natA* and *natB*, *des* and *argI* were specifically up-regulated in the *rasP* mutant. Other transcriptional changes summarized in Table S3A relate to changes in the *sigW* mutant. For the genes that were down-regulated in the *rasP* mutant, most hits were specific for the *rasP* mutant. No clear pattern however emerges from these changes, although some of these genes relate to the cell envelope metabolism (membrane and cell wall). Furthermore, the vast majority of genes found to be differentially expressed in the *prsW* mutant compared to the *sigW* mutant relate to σ^W^-regulated genes. Only the up-regulation of the *pstS*, *pstBA*, *pstBB, pstA* and *pstC* genes for phosphate uptake was very specific for the *prsW* mutant. The reasons for these specific differences in transcription in the *rasP*, *prsW* or *sigW* mutant strains remains to be determined.

## Discussion

The σ^W^-regulon has been extensively described in several previous papers, and 69 genes have been reported as σ^W^-controlled genes [8], [9], [25], [26], [31]. However, it has so far remained very difficult to discriminate between genes of the σ^W^-regulon and the other ECF σ-regulons of *B. subtilis*, as the respective promoter sequences and the stress stimuli for induction partially overlap [9], [14], [25]. Indeed, in the study reporting the transcriptional profile of *B. subtilis* grown in 104 conditions [35], only a global ECF sigma factor regulon was described, and no clear definition of the σ^W^ regulon could be generated. Also, it was so far unknown which ncRNAs of *B. subtilis* are part of the σ^W^-regulon. In our present studies, we have therefore employed tiling array data to define the transcriptome of a *sigW* mutant *B. subtilis* strain. Then the results were examined in the light of the recently described transcriptome of the parental strain 168 across 104 different conditions [35]. Our results show that 89 genes of *B. subtilis* are regulated by σ^W^ and the data suggest that 13–15 of the 69 previously reported σ^W^-regulated genes might represent false-positive identifications. In addition to 53 already known σ^W^-regulated genes, we have discovered 36 novel genes of the σ^W^-regulon and we found that several σ^W^-regulated operons are larger than initially thought.

Two subgroups of σ^W^-regulated genes can be discerned based on the effect values for their down-regulation in *sigW* mutant cells. This differential down-regulation pattern does not correlate with the expression levels of these genes in the parental strain. However, there appears to be a bias for genes that are located at the downstream ends of certain large operons that often have low effect values (i.e. group 2 genes), whereas the genes located more upstream in these operons tend to have high effect values (group 1 genes). On the other hand, several complete operons display high effect values from start to end, while other complete operons have low effect values from start to end. This indicates that the location of a gene in an operon can influence whether it belongs to group 1 or group 2. However, it remains to be determined which additional mechanisms are responsible for the observed bimodal pattern in σ^W^ regulation. Another novel finding was that several apparently non-σ^W^-regulated genes on the opposite strand of σ^W^-regulated genes turned out to be slightly up-regulated in the *sigW* mutant. This indicates that the transcriptional activity of σ^W^-regulated genes can have a negative impact on the transcription of genes encoded by the opposite strand. The molecular basis for this effect is currently not known. However, it is conceivable that RNA-polymerase initiating with σ^W^ may directly or indirectly dampen the transcription elongation efficiency of RNA-polymerase transcribing into the opposite direction.

As expected, the σ^W^-regulated genes were also down-regulated in *rasP* or *prsW* mutant strains, albeit to lesser extents than in the *sigW* mutant. This implies that there is residual σ^W^ activity in the absence of either the RasP or PrsW proteases, which may relate to the equilibrium between the free states of σ^W^ plus RsiW and the σ^W^-RsiW bound state. Such leakiness is not an uncommon feature among biological systems. Alternatively, certain other proteases may also be capable of degrading limited amounts of RsiW in the absence of RasP or PrsW. Candidate proteases for alternative RsiW cleavage in the absence of PrsW might be the membrane-bound forms of HtrA and HtrB. Both HtrA and HtrB are closely related to the site-1 protease DegS of *E. coli*, which has been implicated in RIP of the anti-sigma factor RseA that sequesters σ^E^ [1], [12], [30]. It should be noted that, compared to the previously used methods for assessing the effects of mutations in *rasP* or *prsW* [15], [19], [21], [42], the presently performed tiling array analyses are more sensitive and they can reproducibly reveal smaller changes. This is probably the reason why residual σ^W^ activity in the absence of RasP or PrsW has so far remained unnoticed.

In relation to the previously documented defects of *rasP* mutant cells in competence development [20], [32], protein secretion [32], [41], and membrane protein overproduction [50], we verified whether any of these defects could be connected to transcriptional changes. However, as indicated above, the observed transcriptional changes in the *rasP* mutant were generally very minor and, apart from four competence-related genes, no changes were found that might explain any of the observed phenotypes through transcriptional regulation. The four affected competence-related genes (*nucA*, *oppA*, *ssbB* and *rapD*) were only very slightly down-regulated in the *rasP* mutant and this finding should be viewed with caution, because the present analyses were performed with cells grown in LB medium, which is not an optimal medium for inducing competence. Taken together, we conclude that the observed defects of *rasP* mutant cells in protein secretion and membrane protein overproduction most likely relate to post-transcriptional regulatory mechanisms that would involve the enzymatic activity of the RasP protease. However, we cannot completely exclude the possibility that changes in the membrane fluidity contribute to the pleiotropic phenotype of *rasP* mutant cells. This relates to recent studies by Kingston et al., 2011 [31], who proposed that activation of a σ^W^-dependent promoter in the *fabHa-fabF* operon results in a higher proportion of straight-chain fatty acids and a longer average chain length in phospholipids, which will cause a reduced fluidity of the membrane. It should be noted however that under non-stress conditions we observed no influence of the absence of σ^W^ on the expression of *fabHa*.

In conclusion, the present studies lead to a definition of the σ^W^ regulon under non-stress conditions (exponential growth in LB broth at 37°C) that have been applied in numerous studies over the past decades. Importantly, the use of non-stress conditions allowed us to determine the basal expression levels of σ^W^-regulated genes, and to avoid side effects of particular stresses on the entire regulatory network of the cell. By following this strategy, we have considerably reduced the complexity of the system, which permitted us (i) to pinpoint the most strictly σ^W^-dependent genes that probably have promoter sequences with the highest affinity for σ^W^, and (ii) to classify the known and newly identified σ^W^-controlled genes. Furthermore, our studies provide novel insights in the importance of the RIP proteases PrsW and RasP in the activation of this stress-responsive regulon. Especially, the observation that the absence of either PrsW or RasP does not lead to a complete inactivation of σ^W^-dependent gene expression is intriguing and calls for further investigations. Although this expression is most likely caused by an equilibrium where low levels of σ^W^ bind to RNAP instead of the anti-sigma factor RsiW, it cannot be excluded that certain, so far unknown, signals trigger alternative pathways for RsiW inactivation, or that PrsW and RasP might be substituted to some extent by other proteases. Lastly, our present findings strongly support the view that RasP is not only directly involved in the activation of the σ^W^-regulon, but also in other post-transcriptional regulatory mechanisms relating to competence development, protein secretion and membrane protein biogenesis.

## Supporting Information

Table S1 Previously identified σW-regulated genes.
(XLSX)

Table S2 Genes down- or up-regulated in *sigW*, *prsW* or *rasP* mutant strains. Changes associated with p-values<0.05 are indicated in bold. A, down-regulated genes. B, up-regulated genes.
(DOCX)

Table S3 Genes up- or down-regulated in the *rasP* mutant strain compared to the *sigW* mutant strain. Changes associated with p-values<0.05 are indicated in bold. A, up-regulated genes. B, down-regulated genes.
(DOCX)

Table S4 Genes up- or down-regulated in the *prsW* mutant strain compared to the *sigW* mutant strain. Changes associated with p-values<0.05 are indicated in bold. A, up-regulated genes. B, down-regulated genes.
(DOCX)
