# Supplementary material for: Definition of the σW Regulon of Bacillus subtilis in the Absence of Stress
Source: PLoS One. 2012 Nov 14;7(11):e48471. doi: 10.1371/journal.pone.0048471 (PMC3498285; doi:10.1371/journal.pone.0048471)
Supplement: Table S2 — Genes down- or up-regulated in sigW, prsW or rasP mutant strains. Changes associated with p-values<0.05 are indicated in bold. A, down-regulated genes. B, up-regulated genes. (DOCX) [file pone.0048471.s002.docx]

**Supplementary Table S2. Genes down- or up-regulated in *sigW*, *prsW* or *rasP* mutant strains.**

**Table S2A. Genes down-regulated in *sigW*, *prsW* or *rasP* mutant strains.** Changes associated with p-values < 0.05 are indicated in bold.

| **Name** | **Effect**  ***sigW*/WT** | **Effect *rasP*/WT** | **Effect *prsW*/WT** | **Down** | **Regulation** | **Function** |
| --- | --- | --- | --- | --- | --- | --- |
| *rsiW* | **-6.84** | **-0.53** | **-0.66** | all | σ^W^ | Control of SigW activity |
| *sigW* | **-6.83** | **-0.58** | **-0.76** | all | σ^W^ | Sigma W factor |
| *spo0M* | **-4.92** | **-1.70** | **-1.43** | all | σ^W^ | Sporulation |
| S691 | **-3.66** | **-0.94** | **-1.02** | all | σ^W^ |  |
| *yeaA* | **-3.61** | **-1.30** | **-1.30** | all | Secondary σ^W^ |  |
| *yjoB* | **-3.40** | **-1.17** | **-1.17** | all | σ^W^ |  |
| *ydjP* | **-3.34** | **-1.13** | **-1.22** | all | Secondary σ^W^ |  |
| S462 (indep) | **-3.23** | **-0.89** | **-0.95** | all | σ^W^ |  |
| *yxjI* | **-3.12** | **-0.86** | **-0.95** | all | σ^W^ |  |
| *yoaG* | **-3.07** | **-0.99** | **-0.95** | all | σ^W^ |  |
| *fosB* | **-3.03** | **-1.30** | **-1.35** | all | σ^W^ | Fosfomycin resistance |
| *ythP* | **-2.98** | **-1.27** | **-1.08** | all | σ^W^ | ATP transporter (ATP binding protein) |
| S690 | **-2.90** | **-1.17** | **-1.01** | all | σ^W^ | 3’of *yoaG* |
| S1495 (indep) | **-2.89** | **-1.16** | **-1.19** | all | σ^W^ |  |
| *ythQ* | **-2.74** | **-1.01** | **-1.01** | all | σ^W^ | ABC transporter |
| S742 | **-2.70** | **-0.98** | **-1.77** | all | σ^W^ |  |
| *pspA* | **-2.68** | **-1.21** | **-0.76** | all | Secondary σ^W^ |  |
| *yfhL* | **-2.52** | **-1.16** | **-1.00** | all | Secondary σ^W^ | SdpC resistance |
| *ydjG* | **-2.51** | **-1.03** | **-0.68** | all | secondary σ^W^ |  |
| S719 (inter) | **-2.49** | **-0.97** | **-0.80** | all | σ^W^ |  |
| S658 (inter) | **-2.48** | **-1.09** | **-1.18** | all | σ^W^ |  |
| *ybfO* | **-2.47** | **-1.40** | **-0.78** | all | σ^W^ | Similar to erythromycin esterase |
| *ydbT* | **-2.47** | **-1.00** | **-0.88** | all | Secondary σ^W^ |  |
| *ydbS* | **-2.46** | **-1.04** | **-0.79** | all | Secondary σ^W^ |  |
| *yuaG* | **-2.33** | **-1.32** | **-1.06** | all | σ^W^ | Sporulation (early stage) |
| *pbpE* | **-2.33** | **-1.28** | **-1.20** | all | σ^W^ | Cell wall synthesis |
| *yfhM* | **-2.30** | **-1.02** | **-0.92** | all | Secondary σ^W^ | Survival of ethanol stress |
| *ydjH* | **-2.27** | **-0.93** | **-0.57** | all | Secondary σ^W^ |  |
| *yobJ* | **-2.24** | **-0.75** | **-0.88** | all | σ^W^ |  |
| *ydjI* | **-2.17** | **-0.93** | **-0.61** | all | Secondary σ^W^ |  |
| *racX* | **-2.12** | **-1.25** | **-1.19** | all | σ^W^ | Control of biofilm formation |
| *yuaI* | **-2.02** | **-1.00** | **-1.05** | all | σ^W^ |  |
| *yvlA* | **-1.91** | **-0.46** | **-0.69** | all | σ^W^ |  |
| *yvlB* | **-1.85** | **-0.77** | **-0.67** | all | σ^W^ |  |
| *yuaF* | **-1.78** | **-1.00** | **-1.00** | all | σ^W^ |  |
| *ybfP* | **-1.38** | **-0.78** | **-0.46** | all | σ^W^ |  |
| S89 (inter) | **-1.34** | **-0.74** | **-0.46** | all | σ^W^ |  |
| *yjzH* | **-1.19** | **-0.70** | **-0.67** | all | σ^W^ |  |
| *sppA* | **-1.18** | **-1.16** | **-0.71** | all | Secondary σ^W^ | Signal peptide peptidase |
| *yteJ* | **-1.17** | **-1.10** | **-0.47** | all | Secondary σ^W^ | Resistance against SdpC |
| *yxjH* | **-0.83** | **-0.55** | **-0.48** | all |  |  |
| *yknY* | **-0.76** | **-1.29** | **-0.47** | all | σ^W^ | Resistance against SdpC |
| *yknZ* | **-0.69** | **-1.12** | **-0.42** | all | σ^W^ | Resistance against SdpC |
| *ydjO* | **-0.41** | **-0.40** | **-0.47** | all | Secondary σ^W^ |  |
| *ywrE* | **-1.82** | **-0.40** | **-0.84** | *sigW* and *prsW* | σ^W^ |  |
| S659 (indep) | **-0.94** | **-0.51** | **-0.85** | *sigW* and *prsW* | σ^W^ |  |
| *yozO* | **-0.60** | -0.27 | **-0.43** | *sigW* and *prsW* | σ^W^ |  |
| *ysdB* | **-3.54** | **-1.20** | -1.04 | *sigW* and *rasP* | σ^W^ |  |
| *yqfB* | **-2.25** | **-1.23** | -0.56 | *sigW* and *rasP* | σ^W^ |  |
| *yqfA* | **-2.11** | **-1.09** | -0.37 | *sigW* and *rasP* | σ^W^ |  |
| *yceH* | **-0.88** | **-0.50** | -0.32 | *sigW* and *rasP* | Secondary σ^W^ |  |
| *yceG* | **-0.87** | **-0.42** | -0.34 | *sigW* and *rasP* | Secondary σ^W^ |  |
| *yknX* | **-0.78** | **-1.41** | -0.34 | *sigW* and *rasP* | σ^W^ |  |
| *alsD* | **-0.63** | **-0.70** | -0.47 | *sigW* and *rasP* |  |  |
| *yknW* | **-0.57** | **-1.08** | -0.24 | *sigW* and *rasP* | σ^W^ |  |
| *tatAC* | -0.30 | **-0.72** | **-0.88** | *rasP* and *prsW* |  | Protein secretion |
| S1395 | -0.19 | **-0.71** | **-0.63** | *rasP* and *prsW* |  | 5’ of *ywzD* |
| *ssbB* | -0.12 | **-0.61** | **-0.45** | *rasP* and *prsW* | ComK | Genetic transformation |
| *yjoA* | -0.39 | **-0.58** | **-0.61** | *rasP* and *prsW* |  |  |
| *yjcN* | 0.10 | **-0.50** | **-0.57** | *rasP* and *prsW* | Rok |  |
| *yvfI* | -0.39 | **-0.48** | **-0.46** | *rasP* and *prsW* | LutR | Control of lactate utilization |
| *yqjN* | -0.30 | **-0.42** | **-0.47** | *rasP* and *prsW* |  |  |
| *yqeZ* | **-2.21** | -1.07 | -0.72 | *sigW* | σ^W^ |  |
| *mtlF* | **-2.05** | 0.11 | -0.79 | *sigW* |  |  |
| *mtlD* | **-1.97** | 0.18 | -0.83 | *sigW* |  |  |
| *mtlA* | **-1.85** | 0.01 | -1.01 | *sigW* |  |  |
| *yoaF* | **-1.58** | -0.80 | -0.71 | *sigW* | Secondary σ^W^ |  |
| S160 (inter) | **-1.56** | -0.38 | -0.19 | *sigW* | Secondary σ^W^ |  |
| *yvlD* | **-1.34** | -0.29 | -0.43 | *sigW* | σ^W^ |  |
| *yvlC* | **-1.32** | -0.44 | -0.53 | *sigW* | σ^W^ |  |
| *yaaN* | **-1.11** | -0.58 | -0.45 | *sigW* | σ^W^ |  |
| *yceE* | **-1.04** | -0.52 | -0.46 | *sigW* | Secondary σ^W^ |  |
| S716 | **-0.95** | -0.40 | -0.68 | *sigW* |  |  |
| *yceD* | **-0.90** | -0.46 | -0.47 | *sigW* | Secondary σ^W^ |  |
| S22 (intra) | **-0.88** | -0.28 | -0.16 | *sigW* | σ^W^ |  |
| *yceC* | **-0.84** | -0.45 | -0.35 | *sigW* | secondary σ^W^ |  |
| *ygzA* | **-0.82** | -0.46 | -0.24 | *sigW* |  |  |
| S1338 | **-0.80** | -0.18 | -0.22 | *sigW* | σ^W^ |  |
| S106 | **-0.78** | -0.24 | -0.42 | *sigW* | Secondary σ^W^ |  |
| *ilvD* | **-0.78** | -0.55 | -0.53 | *sigW* |  |  |
| *xpaC* | **-0.77** | -0.23 | -0.49 | *sigW* | σ^W^ |  |
| *yqfC* | **-0.76** | -0.27 | -0.01 | *sigW* |  |  |
| S1175 | **-0.75** | -0.49 | -0.33 | *sigW* |  |  |
| *yceF* | **-0.74** | -0.24 | -0.39 | *sigW* | Secondary σ^W^ |  |
| *yqfD* | **-0.72** | -0.24 | 0.04 | *sigW* |  |  |
| *mtnK* | **-0.65** | -0.40 | -0.23 | *sigW* |  |  |
| S740 (inter) | **-0.54** | -0.11 | -0.04 | *sigW* | Secondary σ^W^ |  |
| S161 | **-0.52** | -0.19 | -0.14 | *sigW* | Secondary σ^W^ |  |
| S739 | **-0.51** | 0.06 | 0.19 | *sigW* | Secondary σ^W^ |  |
| S442 (inter) | **-0.48** | -0.16 | -0.38 | *sigW* | σ^W^ |  |
| S1390 (inter) | **-0.48** | 0.02 | -0.11 | *sigW* | σ^W^ |  |
| *acpS* | **-0.45** | -0.11 | -0.07 | *sigW* | Secondary σ^W^ |  |
| S162 | **-0.44** | -0.07 | 0.05 | *sigW* | Secondary σ^W^ |  |
| *ydcC* | **-0.42** | -0.12 | 0.01 | *sigW* | Secondary σ^W^ |  |
| *thiC* | **-0.41** | -0.20 | -0.22 | *sigW* |  |  |
| *yocM* | **-0.41** | 0.22 | 0.26 | *sigW* | Secondary σ^W^ |  |
| *rasP* | 0.20 | **-6.09** | **0.42** | *rasP* |  | Control of cell division and SigW activity |
| *csn* | -0.35 | **-1.88** | -0.21 | *rasP* | AbrB | Chitin degradation |
| *yxaL* | -0.40 | **-1.46** | -0.82 | *rasP* | AbrB, Rok |  |
| *oppA* | -0.41 | **-1.24** | -0.*2*1 | *rasP* | ScoC, TnrA | Initiation of sporulation, competence development |
| S406 | -0.16 | **-1.18** | -0.22 | *rasP* |  | 5’ of *oppA* |
| *ylqB* | 0.29 | **-1.15** | 0.20 | *rasP* | AbrB, SigD |  |
| *yvyD* | -0.46 | **-1.10** | -0.83 | *rasP* | sigB, sigH | Dimerization of ribosomes in the stationary phase |
| *mreBH* | -0.04 | **-1.09** | 0.02 | *rasP* | sigI | Cell shape determination |
| S935 | 0.15 | **-1.07** | 0.14 | *rasP* |  | 5’ of *yqzD* |
| *yxaJ* | 0.23 | **-1.03** | -0.13 | *rasP* | Rok |  |
| S351 | -0.60 | **-0.96** | -0.49 | *rasP* |  | 3’ of *scoC* |
| *yweA* | 0.12 | **-0.96** | -0.33 | *rasP* |  |  |
| *ykpC* | 0.04 | **-0.94** | 0.05 | *rasP* |  |  |
| *yvzJ* | -0.20 | **-0.92** | -0.83 | *rasP* |  |  |
| S135 | -0.42 | **-0.91** | -0.33 | *rasP* |  | 3’ of *yczJ* |
| S480 | 0.*2*4 | **-0.91** | 0.04 | *rasP* |  | 5’ of *sigI* |
| S1462 | 0.09 | **-0.90** | -0.45 | *rasP* |  | 5’ of *yweA* |
| S407 (inter) | -0.42 | **-0.85** | -0.32 | *rasP* |  | Between *oppA* and *oppB* |
| *ydhK* | -0.12 | **-0.83** | 0.18 | *rasP* | sigB | Survival of ethanol stress |
| *yuaB* | -0.04 | **-0.83** | -0.10 | *rasP* | AbrB, DegU | Pellicle formation |
| *yqxI* | -0.20 | **-0.79** | -0.14 | *rasP* | CcpA, Spo0A |  |
| *yjhA* | -0.12 | **-0.78** | 0.12 | *rasP* |  |  |
| *yttA* | -0.17 | **-0.76** | -0.11 | *rasP* | TnrA |  |
| *wprA* | -0.01 | **-0.75** | -0.06 | *rasP* | YvrHb |  |
| *ptb* | -0.71 | **-0.75** | -0.52 | *rasP* | BkdR, CodY, SigL | Utilization of branched-chain keto acids |
| *lpdV* | -0.41 | **-0.74** | -0.09 | *rasP* | BkdR, CodY, SigL | Utilization of branched-chain keto acids |
| *yolA* | 0.00 | **-0.72** | 0.19 | *rasP* |  |  |
| *bcd* | -0.39 | **-0.72** | -0.30 | *rasP* | BkdR, CodY, SigL | Utilization of branched-chain keto acids |
| *yvbJ* | 0.08 | **-0.72** | -0.04 | *rasP* |  |  |
| *yhbJ* | -0.50 | **-0.70** | -0.02 | *rasP* |  |  |
| *ykvA* | -0.08 | **-0.70** | -0.24 | *rasP* |  |  |
| *yolB* | 0.05 | **-0.70** | 0.15 | *rasP* |  |  |
| *yqzC* | -0.16 | **-0.69** | -0.40 | *rasP* | Spo0A |  |
| *estB* | -0.10 | **-0.68** | -0.33 | *rasP* | AbrB | Lipid degradation |
| *yraL* | -0.29 | **-0.67** | -0.49 | *rasP* |  |  |
| *yxiT/1* | 0.04 | **-0.67** | 0.03 | *rasP* |  |  |
| *serC* | -0.48 | **-0.66** | -0.47 | *rasP* |  | Biosynthesis of serine |
| *purT* | -0.52 | **-0.63** | -0.46 | *rasP* |  | Purine biosynthesis |
| *estA* (*lipA*) | 0.45 | **-0.63** | 0.41 | *rasP* | AbrB | Lipid degradation |
| *bkdAA* | -0.47 | **-0.63** | -0.21 | *rasP* | BkdR, CodY, SigL | Utilization of branched-chain keto acids |
| *yhcB* | -0.22 | **-0.62** | -0.05 | *rasP* |  |  |
| *wapA* | -0.09 | **-0.62** | -0.01 | *rasP* | DegU, YvrHb |  |
| *yhcC* | -0.47 | **-0.61** | -0.14 | *rasP* |  |  |
| *buk* | -0.27 | **-0.61** | -0.07 | *rasP* | BkdR, CodY, SigL | Utilization of branched-chain keto acids |
| *yqzD* | 0.03 | **-0.61** | -0.10 | *rasP* | Spo0A |  |
| *bkdB* | -0.40 | **-0.60** | -0.21 | *rasP* | BkdR, CodY, SigL | Utilization of branched-chain keto acids |
| S1121 | -0.42 | **-0.60** | -0.06 | *rasP* |  | 5’ of *sppA* |
| *lytB* | -0.27 | **-0.60** | -0.27 | *rasP* | LytR, SigD, SlrR, YvrHb | Autolysin (cell wall degradation) |
| *pel* | -0.17 | **-0.58** | -0.40 | *rasP* | CcpA, ComA, TnrA | Degradation of polygalacturonic acid |
| *yhfF* | -0.19 | **-0.58** | -0.13 | *rasP* |  |  |
| *hinT* | -0.30 | **-0.57** | -0.30 | *rasP* |  | Cell-cycle regulation |
| S488 | 0.05 | **-0.57** | -0.27 | *rasP* |  | 5’ of ykvA |
| *yxiT/2* | 0.18 | **-0.57** | 0.13 | *rasP* |  |  |
| *yyzF* | -0.35 | **-0.57** | -0.31 | *rasP* |  |  |
| *nucA* | -0.46 | **-0.56** | -0.49 | *rasP* | ComK | Genetic transformation |
| *htpG* | -0.11 | **-0.56** | -0.23 | *rasP* |  | Chaperone |
| *yxbC* | 0.12 | **-0.56** | 0.00 | *rasP* | AbrB, CodY, Spo0A |  |
| *lytA* | -0.29 | **-0.55** | -0.34 | *rasP* | LytR, SigD, SlrR, YvrHb | Autolysin (cell wall degradation) |
| *yhcA* | -0.34 | **-0.54** | 0.11 | *rasP* |  |  |
| S938 (inter) | 0.00 | **-0.54** | **0.45** | *rasP* |  | Between *yqgA* and *yqgC* |
| S1013 | -0.09 | **-0.54** | -0.40 | *rasP* |  | 5’ of *yraL* |
| *rapD* | 0.00 | **-0.54** | -0.20 | *rasP* | RghR, SigM, SigX | Control of ComA-dependent gene expression |
| *yfnC* | -0.10 | **-0.53** | -0.06 | *rasP* |  |  |
| *ysdC* | -0.26 | **-0.50** | -0.31 | *rasP* |  |  |
| *ycnD* | -0.3*2* | **-0.49** | -0.33 | *rasP* |  | Delivery of FMN to enzymes |
| *yisL* | 0.08 | **-0.47** | -0.38 | *rasP* |  |  |
| *pbpC* | -0.*2*7 | **-0.45** | -0.04 | *rasP* |  | Penicillin binding protein 3 |
| *yuzD* | 0.00 | **-0.45** | -0.34 | *rasP* |  |  |
| *csaA* | -0.27 | **-0.43** | -0.25 | *rasP* |  | Protein secretion |
| *pgi* | -0.26 | **-0.43** | -0.33 | *rasP* |  | Enzyme in glycolysis/gluconeogenesis |
| *ywhB* | -0.19 | **-0.43** | -0.31 | *rasP* |  |  |
| *aprE* | -0.07 | **-0.42** | -0.04 | *rasP* | AbrB, ScoC, SinR | Protein degradation |
| S937 | 0.12 | **-0.42** | 0.34 | *rasP* |  | 5’ of *yqgC* |
| *lytC* | -0.14 | **-0.42** | -0.15 | *rasP* | LytR, SigD, SlrR, YvrHb | Autolysin (cell wall degradation) |
| *xynA* | 0.24 | **-0.41** | -0.18 | *rasP* |  | Xylan degradation |
| *prsW* | 0.19 | 0.27 | **-4.30** | *prsW* |  | Control of SigW activity |
| S198 (indep) | -0.57 | -0.47 | **-1.71** | *prsW* |  |  |
| NA | -0.14 | -0.30 | **-1.22** | *prsW* |  |  |
| *rocA* | -0.68 | **1.40** | **-1.06** | *prsW* | AhrC, CodY, RocR, SigL |  |
| S718 (indep) | -0.56 | 0.10 | **-0.92** | *prsW* |  |  |
| *ylaF* | 0.18 | -0.29 | **-0.90** | *prsW* |  |  |
| NA | -0.04 | -0.19 | **-0.87** | *prsW* |  |  |
| S415 (indep) | 0.14 | -0.15 | **-0.86** | *prsW* |  |  |
| *yjzD* | -0.13 | -0.29 | **-0.76** | *prsW* |  |  |
| *phrF* | **-0.18** | -0.18 | **-0.60** | *prsW* | ComA, SigH | Control of ComA activity |
| S1138 | -0.03 | -0.16 | **-0.59** | *prsW* |  | 3’ of *tyrS* |
| *yobD* | -0.06 | -0.37 | **-0.58** | *prsW* |  |  |
| *ytzK* | 0.04 | -0.24 | **-0.57** | *prsW* |  |  |
| S1406 | -0.19 | -0.10 | **-0.57** | *prsW* |  | 5’ of *ywnH* |
| *yqgX* | 0.03 | -0.19 | **-0.48** | *prsW* |  |  |
| S1308 | -0.36 | -0.33 | **-0.45** | *prsW* |  | 5’ of *yvfI* |
| *rplF* | -0.34 | -0.25 | **-0.44** | *prsW* |  | Ribosomal protein |
| *rpsE* | -0.19 | -0.15 | **-0.42** | *prsW* |  | Ribosomal protein |
| *deoR* | -0.18 | -0.15 | **-0.42** | *prsW* | DeoR | Regulation of deoxyribonucleotide utilization |

**Table S2B. Genes up-regulated in *sigW*, *prsW* or *rasP* mutant strains.** Changes associated with p-values < 0.05 are indicated in bold.

| **Name** | **Effect *sigW*/WT** | **Effect**  ***rasP*/WT** | **Effect**  ***prsW*/WT** | **up** | **Regulation** | **function** |
| --- | --- | --- | --- | --- | --- | --- |
| S1026 (inter) | **0.92** | **0.59** | **0.96** | all |  |  |
| S981 (3’) | **0.73** | **0.59** | **0.45** | all |  |  |
| S118 (inter) | **0.52** | **0.66** | **0.75** | all |  | Opposite of *yuaI*-*yuaF-yuaG* |
| *obg* | **0.50** | **0.65** | **1.00** | all |  | Ribosome assembly (essential), possibly required for Spo0A activation |
| *yrzI* | **0.63** | 0.34 | **0.76** | *sigW* and *prsW* | AbrB |  |
| *yktD* | **0.43** | 0.37 | **0.60** | *sigW* and *prsW* |  |  |
| S1380 | **1.16** | **1.55** | 0.73 | *sigW* and *rasP* |  | 5’ of *ywtF* |
| *ydeH* | **0.56** | **0.53** | 0.42 | *sigW* and *rasP* | AbrB |  |
| S303 | **0.45** | **0.60** | 0.35 | *sigW* and *rasP* |  | 5’ of *ygxA* |
| S663 | **0.61** | **0.52** | 0.35 | *sigW* and *rasP* |  | 5’ of *ccdA* |
| S1356 | **0.64** | **0.70** | -0.13 | *sigW* and *rasP* |  | 5’ of *degS* |
| S862 | **0.65** | **0.64** | 0.41 | *sigW* and *rasP* |  | 5’ of *spoIVA* |
| *murG* | **0.77** | **1.08** | 0.56 | *sigW* and *rasP* |  | Peptidoglycan precursor biosynthesis |
| *ywkF* | 0.36 | **0.88** | **0.83** | *prsW* and *rasP* |  | Sporulation protein |
| S143 (3’PT) | 0.43 | **0.73** | **0.79** | *prsW* and *rasP* |  |  |
| *tagH* | 0.40 | **0.69** | **0.70** | *prsW* and *rasP* |  | Biosynthesis of teichoic acid |
| *aapA* | 0.39 | **0.66** | **0.57** | *prsW* and *rasP* |  | Amino acid uptake |
| S1166 (inter) | 0.32 | **0.65** | **0.79** | *prsW* and *rasP* |  | Between *metK* and *asnB* |
| S280 | 0.38 | **0.62** | **0.57** | *prsW* and *rasP* |  | 5’ of *dusC* |
| *rsmE* (*yqeU*) | 0.46 | **0.62** | **0.64** | *prsW* and *rasP* | HrcA |  |
| *spo0B* | 0.32 | **0.60** | **0.75** | *prsW* and *rasP* |  | Initiation of sporulation |
| *dusC* | 0.29 | **0.57** | **0.54** | *prsW* and *rasP* |  | tRNA modification |
| *prmA* (*yqeT*) | 0.41 | **0.56** | **0.60** | *prsW* and *rasP* | HrcA |  |
| *spcB* (*scpB*) | 0.28 | **0.54** | **0.50** | *prsW* and *rasP* |  | Maintenance of chromosome structure |
| *yttB* | 0.36 | **0.54** | **0.54** | *prsW* and *rasP* |  | “resistance agains toxins”? |
| *yszB* (*pheB*) | 0.37 | **0.53** | **0.86** | *prsW* and *rasP* |  | Biosynthesis of phenyl alanine |
| *recJ* (*yrvE*) | 0.35 | **0.51** | **0.55** | *prsW* and *rasP* |  | DNA repair/recombination |
| *ywqB* | 0.40 | **0.50** | **0.59** | *prsW* and *rasP* |  |  |
| *ycnC* | 0.20 | **0.46** | **0.71** | *prsW* and *rasP* |  | “control of transcription factors” |
| *pheA* | 0.38 | **0.44** | **0.76** | *prsW* and *rasP* |  | Biosynthesis of phenyl alanine |
| *speD* | 0.39 | **0.42** | **0.46** | *prsW* and *rasP* | ccpN | Spermidine/polyamine biosynthesis |
| *opuBD* | 0.22 | **0.42** | **0.49** | *prsW* and *rasP* |  | Compatible solute transport (choline) |
| S1355 (intra) | 0.31 | **0.41** | **0.40** | *prsW* and *rasP* |  | Between *degS* and *degU* |
| *ybbK* | **3.07** | -0.69 | -0.54 | *sigW* |  | Opposite of *sigW* |
| *ybbJ* | **2.68** | -0.61 | -0.70 | *sigW* |  | Opposite of *sigW* |
| S928 (inter) | **2.25** | -0.59 | -0.41 | *sigW* |  | Between *mgsR* and *rsbRD* |
| *ykzV* | **1.13** | -0.31 | 0.05 | *sigW* |  |  |
| *cotT* | **0.91** | -0.40 | -0.49 | *sigW* | sigK | Spore coat protein |
| *yodI* | **0.83** | -0.14 | 0.40 | *sigW* | sigK |  |
| S1030 | **0.82** | 0.65 | 0.00 | *sigW* |  | 5’ of *yrhF* |
| *ymaG* | **0.68** | 0.08 | 0.20 | *sigW* | sigK | Spore coat protein |
| S655 | **0.66** | 0.18 | 0.28 | *sigW* |  | Opposite of *fosB* |
| S613 | **0.62** | 0.27 | 0.45 | *sigW* |  | 5’ of *ymzD* |
| S254 (indep) | **0.60** | -0.07 | 0.05 | *sigW* |  |  |
| S1405 (inter) | **0.60** | -0.17 | 0.05 | *sigW* |  |  |
| *ykzW* | **0.59** | 0.08 | -0.11 | *sigW* | CcpN | RNA that inhibits AhrC translation |
| S653 (indep) | **0.57** | 0.25 | 0.38 | *sigW* |  | 5’ of *ccdA* |
| S360 (inter) | **0.54** | -0.06 | 0.23 | *sigW* |  | Between *yhfC* and *yhfE* |
| *yqaR* | **0.54** | 0.21 | -0.18 | *sigW* |  |  |
| *cotU* | **0.50** | 0.28 | 0.17 | *sigW* |  | Spore coat protein |
| *pssA* | **0.46** | 0.28 | 0.33 | *sigW* |  | Biosynthesis of phospholipids |
| S278 | **0.46** | -0.08 | 0.13 | *sigW* |  | 5’ of *yfzA* |
| *yqxD* | **0.46** | 0.24 | 0.38 | *sigW* | sigH |  |
| *comK* | **0.44** | 0.17 | -0.05 | *sigW* | AbrB, ComK, DegU, CodY, Rok | Competence and DNA uptake regulation |
| S1543 (intra) | **0.43** | 0.17 | 0.11 | *sigW* |  | Between *yydH* and *yydI* |
| S95 | **0.42** | 0.20 | 0.09 | *sigW* |  | 5’ of *ycbJ* |
| S427 | **0.42** | -0.06 | -0.14 | *sigW* |  | 5’ of *ypeP* |
| S831 | **0.42** | 0.08 | 0.27 | *sigW* |  | 5’ of *yjzE* |
| *yfzA* | **0.41** | -0.03 | -0.02 | *sigW* |  |  |
| S924 | **0.41** | -0.08 | -0.23 | *sigW* |  | 5’ of *sinI* |
| S825 | 0.73 | **1.72** | 0.59 | *rasP* |  | 5’ of *metA* |
| *des* | 0.32 | **1.69** | 0.21 | *rasP* | DesR | Phospholipid desaturase, reguation of membrane fluidity at low temperatures |
| *natA* | 0.15 | **1.64** | 0.15 | *rasP* | NatR | Sodium export |
| *natB* | 0.03 | **1.57** | 0.00 | *rasP* | NatR | Sodium export |
| *rocA* | -0.68 | **1.40** | -1.06 | *rasP* | AhrC, CodY, RocR, sigL | Arginine, ornithin and citrullin utilization |
| S1023 | 0.42 | **1.33** | 0.44 | *rasP* |  |  |
| *rocD* | -0.39 | **1.30** | -0.28 | *rasP* | AhrC, CodY, RocR, sigL, Spo0A | Arginine, ornithin and citrullin utilization |
| S1551 (intra) | -0.37 | **1.28** | -0.22 | *rasP* |  | Between *rocD* and *rocE* |
| *rocE* | -0.15 | **1.20** | -0.06 | *rasP* | AhrC, CodY, RocR, sigL, Spo0A | Arginine, ornithin and citrullin utilization |
| S881 (intra) | 0.38 | **1.18** | 0.79 | *rasP* |  | Between *ypuD* and *ribD* |
| *yebC* | 0.27 | **1.09** | 0.49 | *rasP* | sigM |  |
| *yqjL* | 0.37 | **1.05** | 0.37 | *rasP* | sigB, sigM, sigW | Resistance against paraquat |
| *hisB* | 0.16 | **1.05** | 0.25 | *rasP* |  | Biosynthesis of histidine |
| *sirC* (*ylnF*) | 0.45 | **1.03** | 0.91 | *rasP* | CymR, S-box | Siroheme synthesis, sulfite reduction |
| *argI* (*rocF*) | -0.02 | **1.02** | -0.10 | *rasP* | AhrC, CodY, RocR, sigL, Spo0A | Arginine utilization |
| *sirB* (*ylnE*) | 0.53 | **1.01** | 0.94 | *rasP* | CymR, S-box | Siroheme synthesis, sulfite reduction |
| *hisD* | 0.05 | **1.01** | 0.11 | *rasP* |  | Histidine biosynthesis |
| *rocB* | -0.29 | **0.99** | -0.26 | *rasP* | AhrC, CodY, RocR, sigL | Arginine, ornithin and citrullin utilization |
| *yuiF* | 0.37 | **0.97** | 0.24 | *rasP* |  |  |
| *trnD-*Trp | 0.76 | **0.95** | 0.24 | *rasP* |  | Transfer RNA Trp |
| S1270 (3’MT) | 0.25 | **0.95** | 0.12 | *rasP* |  | 3’MT of *yuzO* |
| *hisF* | 0.03 | **0.95** | 0.14 | *rasP* |  | Biosynthesis of histidine |
| *lmrA* | 0.61 | **0.94** | 0.72 | *rasP* | LmrA | Regulation of lincomycin resistance |
| S250 | 0.34 | **0.93** | 0.57 | *rasP* |  | 5’ of *ltaSA* |
| *liaI* | 0.16 | **0.92** | 0.29 | *rasP* | LiaR | Resistance against stress and cell wall antibiotics |
| *trnI-*Thr | 0.47 | **0.91** | 0.26 | *rasP* |  | Transfer RNA Thr |
| *hisH* | 0.04 | **0.91** | 0.22 | *rasP* |  | Biosynthesis of histidine |
| S1332 | 0.42 | **0.91** | 0.16 | *rasP* |  | 5’ of *hisZ* |
| S247 | 0.33 | **0.90** | 0.32 | *rasP* |  |  |
| *hisG* | 0.12 | **0.90** | 0.17 | *rasP* |  | Biosynthesis of histidine |
| *rodA* | 0.54 | **0.90** | 0.47 | *rasP* | sigM | Control of cell shape and elongation |
| *yocC* | 0.33 | **0.89** | 0.48 | *rasP* |  |  |
| *ydaH* | 0.28 | **0.88** | 0.39 | *rasP* | sigM |  |
| *fabHB* | 0.33 | **0.88** | 0.59 | *rasP* | FapR | Fatty acid biosynthesis |
| *hisI* | -0.07 | **0.87** | 0.06 | *rasP* |  | Biosynthesis of histidine |
| *hisA* | 0.00 | **0.86** | 0.12 | *rasP* |  | Biosynthesis of histidine |
| *yccK* | 0.15 | **0.85** | 0.18 | *rasP* |  | Downstream of *natAB* |
| NA | 0.29 | **0.85** | 0.69 | *rasP* |  |  |
| S48 (intra) | 0.37 | **0.84** | 0.43 | *rasP* |  | Between *secE* and *nusG* |
| S620 | 0.54 | **0.84** | 0.33 | *rasP* |  | 3’of *ymzA* |
| S843 | 0.58 | **0.83** | 0.73 | *rasP* |  |  |
| S1581 (inter) | 0.43 | **0.81** | 0.74 | *rasP* |  | Between *rpmH* and *rnpA* |
| *ktrD* | 0.45 | **0.80** | 0.42 | *rasP* |  | Potassium uptake |
| S1368 | 0.33 | **0.80** | 0.10 | *rasP* |  |  |
| *yczE* | 0.30 | **0.79** | 0.22 | *rasP* |  |  |
| *hisZ* | 0.15 | **0.77** | 0.11 | *rasP* |  | translation |
| *prmC* (*ywkE*) | 0.19 | **0.77** | 0.73 | *rasP* |  |  |
| S621 (inter) | 0.45 | **0.75** | 0.39 | *rasP* |  | Between *ymzA* and *nrdI* |
| *tagG* | 0.37 | **0.74** | 0.47 | *rasP* |  | Teichoic acid synthesis |
| *opuAB* | 0.08 | **0.73** | 0.26 | *rasP* |  | Compatible solute transport (glycine betaine) |
| NA | 0.43 | **0.70** | 0.24 | *rasP* |  |  |
| *ypbG* | 0.06 | **0.67** | 0.20 | *rasP* | sigM |  |
| S426 | 0.26 | **0.66** | 0.31 | *rasP* |  | 5’of *yjcD* |
| *yonP* | -0.02 | **0.66** | 0.08 | *rasP* |  | SPβ prophage |
| S255 | 0.39 | **0.65** | 0.27 | *rasP* |  |  |
| S270 | 0.18 | **0.65** | 0.51 | *rasP* |  | 5’of *yflE* |
| *yhdK* | 0.18 | **0.65** | 0.56 | *rasP* | sigM | Anti-σ^M^-factor |
| *desK* | 0.13 | **0.65** | 0.31 | *rasP* | DesR | Control of des-expression |
| *radC* | 0.34 | **0.65** | 0.39 | *rasP* | comK, sigM | DNA repair |
| *liaH* | -0.03 | **0.65** | 0.22 | *rasP* | LiaR | Resistance against stress and cell wall antibiotics |
| S1428 (inter) | 0.29 | **0.64** | 0.37 | *rasP* |  | Between *ywlG* and *glyA* |
| S459 | 0.37 | **0.63** | 0.48 | *rasP* |  | 5’ of *ykaA* |
| S290 (inter) | 0.38 | **0.62** | 0.42 | *rasP* |  | Between *yfhI* and *yfhJ* |
| *cgeD* | 0.01 | **0.62** | -0.05 | *rasP* | GerE, sigK | Sporulation |
| *yqkK* | 0.29 | **0.62** | 0.16 | *rasP* |  |  |
| S1532 | 0.22 | **0.61** | 0.21 | *rasP* |  | 5’of *yxbF* |
| S144 (indep) | -0.07 | **0.60** | 0.24 | *rasP* |  |  |
| *yneJ* | 0.42 | **0.60** | 0.27 | *rasP* | sigA |  |
| *yxjN* | 0.23 | **0.60** | 0.19 | *rasP* |  |  |
| S1340 | 0.24 | **0.58** | 0.09 | *rasP* |  | 5’of *yvlA* |
| *thiD* | 0.10 | **0.57** | 0.22 | *rasP* | Thi-box | Biosynthesis of thiamine pyrophosphate |
| S916 | 0.43 | **0.57** | 0.51 | *rasP* |  | 5’of *dxs* |
| S1046 | 0.25 | **0.56** | 0.27 | *rasP* |  | 5’of *yrvC* |
| *ypzH* | 0.13 | **0.54** | 0.14 | *rasP* |  |  |
| *yopE* | 0.37 | **0.53** | 0.29 | *rasP* |  | SPβ-prophage |
| *menH* | 0.35 | **0.53** | 0.52 | *rasP* |  | Menaquinone biosynthesis (essential) |
| *yhdL* | 0.21 | **0.52** | 0.38 | *rasP* | sigM | Anti-σ^M^-factor |
| S662 (inter) | 0.41 | **0.51** | 0.48 | *rasP* |  | Between *yneF* and *ccdA* |
| *ykuC* | 0.24 | **0.50** | 0.38 | *rasP* |  |  |
| S1093 | 0.39 | **0.50** | 0.39 | *rasP* |  | 5’ of *speD* |
| *opuAC* | -0.15 | **0.48** | 0.17 | *rasP* |  | Compatible solute transport (glycine betaine) |
| S1231 | 0.29 | **0.48** | 0.03 | *rasP* |  | 5’ of *yuiH* |
| *yueF* | 0.11 | **0.47** | 0.32 | *rasP* |  |  |
| *yhbA* | 0.28 | **0.46** | 0.43 | *rasP* |  | Biosynthesis of queuosine, tRNA modification |
| *ykvT* | 0.25 | **0.46** | 0.28 | *rasP* | WalR |  |
| *sigM* | 0.08 | **0.45** | 0.19 | *rasP* | sigM | Resistance against cell envelope stress, oxidative stress and salt stress |
| *ywoG* | 0.30 | **0.45** | 0.37 | *rasP* |  |  |
| *ykuD* | 0.09 | **0.44** | 0.35 | *rasP* | ccpA, sigK | Cell wall biosynthesis |
| *ykuE* | 0.10 | **0.44** | 0.24 | *rasP* |  |  |
| *pgsA* | 0.24 | **0.44** | 0.35 | *rasP* |  | Biosynthesis of phospholipids |
| *clsA* | 0.08 | **0.44** | 0.18 | *rasP* |  | Biosynthesis of phospholipids |
| *yetH* | 0.21 | **0.42** | 0.27 | *rasP* |  |  |
| *ymaC* | 0.15 | **0.42** | 0.30 | *rasP* |  |  |
| *desR* | 0.00 | **0.42** | 0.28 | *rasP* | DesR | Control of des-expression |
| *yuiH* | 0.28 | **0.42** | 0.02 | *rasP* |  |  |
| *spmA* | 0.15 | **0.41** | 0.40 | *rasP* | sigE | Germination |
| *yqxC* | 0.24 | **0.41** | 0.33 | *rasP* |  |  |
| *yxbF* | 0.02 | **0.41** | 0.03 | *rasP* |  |  |
| *yetN* | 0.09 | **0.40** | 0.16 | *rasP* |  |  |
| *pstS* | -0.67 | -0.59 | **1.71** | *prsW* | PhoP | High affinity phosphate uptake |
| *opuCD* | 0.35 | 0.84 | **1.19** | *prsW* |  | Compatible solute transport (glycine betaine, carnithine, choline transport) |
| *opuCC* | 0.22 | 0.74 | **1.13** | *prsW* |  | Compatible solute transport (glycine betaine, carnithine, choline transport) |
| *opuCB* | 0.19 | 0.71 | **1.00** | *prsW* |  | Compatible solute transport (glycine betaine, carnithine, choline transport) |
| *phrH* | 0.55 | 0.29 | **0.85** | *prsW* | RghR | Control of sporulation initiation |
| S527 (inter) | 0.39 | 0.16 | **0.83** | *prsW* |  | Between *abh* and *kinC* |
| S1059 (inter) | 0.41 | 0.33 | **0.78** | *prsW* |  | Between *rpmA* and *spo0B* |
| *mlpA* | 0.51 | 0.39 | **0.71** | *prsW* |  | Function of proteolytic activity |
| *rapH* | 0.33 | 0.14 | **0.70** | *prsW* | RghR, ComK | Control of sporulation and ComA activity |
| *maeA* | 0.17 | 0.20 | **0.70** | *prsW* | MalR | Malate utilization |
| S589 (5’) | 0.37 | 0.57 | **0.69** | *prsW* |  | 5’ of *uppS* |
| S787 (inter) | 0.22 | 0.12 | **0.69** | *prsW* |  | Between *yopJ* and *yopK* |
| *psd* | 0.43 | 0.39 | **0.68** | *prsW* | sigX | Phospholipid biosynthesis |
| *ylxW* | 0.27 | 0.51 | **0.67** | *prsW* | sigE, sigM, SpoIIID | Downstream of *divIC* |
| S840 (inter) | 0.25 | 0.10 | **0.66** | *prsW* |  | Between *yprB* and *cotD* |
| *ykyB* | 0.22 | 0.26 | **0.61** | *prsW* |  |  |
| *tagE* | 0.21 | 0.38 | **0.60** | *prsW* | PhoP, WalR | Biosynthesis of teichoic acids |
| *yetK* | 0.18 | 0.29 | **0.59** | *prsW* |  |  |
| *ylxY* | 0.27 | 0.13 | **0.59** | *prsW* |  |  |
| S1283 (intra) | -0.01 | 0.14 | **0.58** | *prsW* |  | Between *copZ* and *copA* |
| *opuBA* | 0.32 | 0.30 | **0.56** | *prsW* | sigA | Compatible solute transport (choline) |
| *yvnB* | 0.13 | 0.12 | **0.56** | *prsW* |  |  |
| S297 | 0.20 | 0.35 | **0.55** | *prsW* |  | 5’ of *yfhQ* |
| *yugK* | -0.05 | 0.22 | **0.54** | *prsW* |  |  |
| S50 (intra) | 0.14 | 0.30 | **0.53** | *prsW* |  | Between *rplK* and *rplA* |
| S558 (intra) | 0.30 | 0.23 | **0.53** | *prsW* |  | Between *murE* and *mraY* |
| S252 (inter) | 0.15 | 0.02 | **0.52** | *prsW* |  | Between *yfmI* and *yfmK* |
| *yvzA* | 0.07 | 0.42 | **0.52** | *prsW* | AbrB, DegU |  |
| *recG* | 0.14 | 0.18 | **0.49** | *prsW* | AbrB, DegU | DNA repair and chromosomal segregation |
| *mreB* | 0.20 | 0.36 | **0.49** | *prsW* | sigM | Cell shape determination |
| *yrvM* | 0.11 | 0.39 | **0.48** | *prsW* |  |  |
| *ydiP* | 0.16 | 0.35 | **0.47** | *prsW* | LexA | BsuM modification (essential) |
| *dtd* | 0.19 | 0.18 | **0.46** | *prsW* |  | Putative D-Tyr-tRNA-(tyr) deacylase |
| *ygxA* | 0.26 | 0.38 | **0.45** | *prsW* |  |  |
| *yqfL* | 0.33 | 0.20 | **0.44** | *prsW* |  | Inhibits CcpN activity |
| *ytqA* | 0.24 | 0.40 | **0.44** | *prsW* |  |  |
| *ylbE* | 0.00 | 0.17 | **0.43** | *prsW* | sigK |  |
| *yopB* | 0.08 | 0.06 | **0.43** | *prsW* |  | SPβ prophage |
| *sul* | -0.10 | 0.09 | **0.42** | *prsW* |  | Folate biosynthesis |
| *ydiC* | 0.17 | 0.32 | **0.42** | *prsW* |  | (essential) |
| *rnhB* | 0.12 | 0.25 | **0.42** | *prsW* |  | Endonucleolytic cleavage of RNA in RNA-DNA hybrid molecules |
| *rseP* | 0.20 | -6.09 | **0.42** | *prsW* |  | Activation of *sigW* |
| *ynbA* | 0.13 | 0.23 | **0.42** | *prsW* |  | GTP-binding protein |
| *rimI* (*ydiD*) | 0.18 | 0.37 | **0.41** | *prsW* |  |  |
