# Supplementary material for: Definition of the σW Regulon of Bacillus subtilis in the Absence of Stress
Source: PLoS One. 2012 Nov 14;7(11):e48471. doi: 10.1371/journal.pone.0048471 (PMC3498285; doi:10.1371/journal.pone.0048471)
Supplement: Table S3 — Genes up- or down-regulated in the rasP mutant strain compared to the sigW mutant strain. Changes associated with p-values<0.05 are indicated in bold. A, up-regulated genes. B, down-regulated genes. (DOCX) [file pone.0048471.s003.docx]

**Supplementary Table S3. Genes up- or down-regulated in the *rasP* mutant strain compared to the *sigW* mutant strain.**

**Table S3A. Genes up-regulated in the *rasP* mutant strain compared to the *sigW* mutant strain.** Changes associated with p-values < 0.05 are indicated in bold.

| **Name** | ***rasP*/WT** | ***sigW*/WT** | ***rasP*/*sigW*** | **σ^W^** | **Function** |
| --- | --- | --- | --- | --- | --- |
| *rsiW* | **-0.53** | **-6.84** | **6.31** | σ^W^ | Control of SigW activity |
| *sigW* | **-0.58** | **-6.83** | **6.25** | σ^W^ | Sigma W factor |
| *spo0M* | **-1.70** | **-4.92** | **3.22** | σ^W^ | Sporulation |
| S691 | **-0.94** | **-3.66** | **2.72** | σ^W^ |  |
| S462 (indep) | **-0.89** | **-3.23** | **2.34** | σ^W^ |  |
| *ysdB* | **-1.20** | **-3.54** | **2.34** | σ^W^ |  |
| *yeaA* | **-1.30** | **-3.61** | **2.31** | σ^W^ |  |
| *yxjI* | **-0.86** | **-3.12** | **2.26** | σ^W^ |  |
| *yjoB* | **-1.17** | **-3.40** | **2.23** | σ^W^ |  |
| *ydjP* | **-1.13** | **-3.34** | **2.21** | σ^W^ |  |
| *mtlF* | 0.11 | **-2.05** | **2.16** |  | Uptake of mannitol |
| *mtlD* | 0.18 | **-1.97** | **2.15** |  | Uptake of mannitol |
| *yoaG* | **-0.99** | **-3.07** | **2.08** | σ^W^ |  |
| *rocA* | **1.40** | -0.68 | **2.08** |  | Arginine, citrulline and ornithine uptake |
| *ythQ* | **-1.01** | **-2.74** | **1.73** | σ^W^ | ABC transporter |
| *fosB* | **-1.30** | **-3.03** | **1.73** | σ^W^ | Fosfomycin resistance |
| S1495 | **-1.16** | **-2.89** | **1.73** | σ^W^ |  |
| S742 | **-0.98** | **-2.70** | **1.73** | σ^W^ |  |
| S690 | **-1.17** | **-2.90** | **1.72** | σ^W^ |  |
| *ythP* | **-1.27** | **-2.98** | **1.71** | σ^W^ | ATP transporter (ATP binding protein) |
| *rocD* | **1.30** | -0.39 | **1.69** |  | Arginine, ornithin and citrullin utilization |
| S1551 | **1.28** | -0.37 | **1.65** |  | Between *rocD* and *rocE* |
| S67 | 0.78 | -0.84 | **1.63** |  | Between *rsiW* and *ybbK* |
| *natB* | **1.57** | 0.03 | **1.54** |  | Sodium export |
| S719 | **-0.97** | **-2.49** | **1.52** | σ^W^ |  |
| *natA* | **1.64** | 0.15 | **1.50** |  | Sodium export |
| *yobJ* | **-0.75** | **-2.24** | **1.49** | σ^W^ |  |
| *ydjG* | **-1.03** | **-2.51** | **1.48** | σ^W^ |  |
| *pspA* | **-1.21** | **-2.68** | **1.47** | σ^W^ |  |
| *ydbT* | **-1.00** | **-2.47** | **1.46** | σ^W^ |  |
| *yvlA* | **-0.46** | **-1.91** | **1.45** | σ^W^ |  |
| *ywrE* | -0.40 | **-1.82** | **1.42** | σ^W^ |  |
| *ydbS* | **-1.04** | **-2.46** | **1.42** | σ^W^ |  |
| S658 | **-1.09** | **-2.48** | **1.39** | σ^W^ |  |
| *des* | **1.69** | 0.32 | **1.37** |  | Phospholipid desaturase, reguation of membrane fluidity at low temperatures |
| *yfhL* | **-1.16** | **-2.52** | **1.36** | σ^W^ | ScpC resistance |
| *rocE* | **1.20** | -0.15 | **1.35** |  | Arginine, ornithin and citrullin utilization |
| *ydjH* | **-0.93** | **-2.27** | **1.34** | σ^W^ |  |
| *rocB* | **0.99** | -0.29 | **1.28** |  | Arginine, ornithin and citrullin utilization |
| *yfhM* | **-1.02** | **-2.30** | **1.28** | σ^W^ | Survival of ethanol stress |
| *ydjI* | **-0.93** | **-2.17** | **1.25** | σ^W^ |  |
| S160 | -0.38 | **-1.56** | **1.17** | σ^W^ |  |
| *yvlB* | **-0.77** | **-1.85** | **1.09** | σ^W^ |  |
| *ybfO* | **-1.40** | **-2.47** | **1.08** | σ^W^ | Similar to erythromycin esterase |
| *yvlD* | -0.29 | **-1.34** | **1.05** | σ^W^ |  |
| *pbpE* | **-1.28** | **-2.33** | **1.05** | σ^W^ | Cell wall synthesis |
| *argI* | **1.02** | **-**0.02 | **1.05** |  | Arginine utilization |
| *yuaI* | **-1.00** | **-2.02** | **1.02** | σ^W^ |  |
| *yuaG* | **-1.32** | **-2.33** | **1.01** | σ^W^ | Sporulation (early stage) |

**Table S3B. Genes down-regulated in the *rasP* mutant strain compared to the *sigW* mutant strain** Changes associated with p-values < 0.05 are indicated in bold.

| **Name** | ***rasP*/WT** | ***sigW*/WT** | ***rasP*/*sigW*** | **Function** |
| --- | --- | --- | --- | --- |
| *rasP* | **-6.09** | 0.20 | **-6.29** | Control of cell division and SigW activity |
| *ybbK* | -0.69 | **3.07** | **-3.76** | Opposite of *sigW* |
| *ybbJ* | -0.61 | **2.68** | **-3.29** | Opposite of *sigW* |
| S928 | -0.59 | **2.25** | **-2.84** | Between *mgsR* and *rsbRD* |
| *murQ* | -1.23 | 0.91 | **-2.14** | Cell wall turnover |
| *ybbH* | -0.98 | 0.94 | **-1.91** | Transcriptional regulator |
| *spbC* | -0.70 | 1.11 | **-1.80** | Toxin, kills non-sporulating cells |
| *csn* | -1.88 | -0.35 | **-1.54** | Chitin degradation |
| *ylqB* | -1.15 | 0.29 | **-1.45** |  |
| *ykzV* | -0.31 | **1.13** | **-1.44** |  |
| *cotT* | -0.40 | **0.91** | **-1.31** | Spore coat protein |
| *yxaJ* | -1.03 | 0.23 | **-1.25** |  |
| S935 | -1.07 | 0.15 | **-1.22** |  |
| S480 | -0.91 | 0.24 | **-1.15** |  |
| *estA* | -0.63 | 0.45 | **-1.09** | Extracellular lipase |
| *yweA* | -0.96 | 0.12 | **-1.08** |  |
| *mreBH* | **-1.09** | -0.04 | **-1.06** | Cell shape determination |
