# Supplementary material for: Definition of the σW Regulon of Bacillus subtilis in the Absence of Stress
Source: PLoS One. 2012 Nov 14;7(11):e48471. doi: 10.1371/journal.pone.0048471 (PMC3498285; doi:10.1371/journal.pone.0048471)
Supplement: Table S4 — Genes up- or down-regulated in the prsW mutant strain compared to the sigW mutant strain. Changes associated with p-values<0.05 are indicated in bold. A, up-regulated genes. B, down-regulated genes. (DOCX) [file pone.0048471.s004.docx]

**Supplementary Table S4. Genes up- or down-regulated in the *prsW* mutant strain compared to the *sigW* mutant strain.**

**Table S4A. Genes up-regulated in the *prsW* mutant strain compared to the *sigW* mutant strain.** Changes associated with p-values < 0.05 are indicated in bold.

| **Name** | ***prsW*/WT** | ***sigW*/WT** | ***prsW*/*sigW*** | **σ^W^** | **Function** |
| --- | --- | --- | --- | --- | --- |
| *rsiW* | **-0.66** | **-6.84** | **6.18** | σ^W^ | Control of SigW activity |
| *sigW* | **-0.76** | **-6.83** | **6.07** | σ^W^ | Sigma W factor |
| *spo0M* | **-1.43** | **-4.92** | **3.49** | σ^W^ | Sporulation |
| S691 | **-1.02** | **-3.66** | **2.64** | σ^W^ |  |
| *ysdB* | -1.04 | **-3.54** | **2.49** | σ^W^ |  |
| *pstS* | **1.71** | -0.67 | **2.38** |  | Phosphate ABC transporter (binding protein) |
| *yeaA* | **-1.30** | **-3.61** | **2.31** | σ^W^ |  |
| S462 | **-0.95** | **-3.23** | **2.28** | σ^W^ |  |
| *yjoB* | **-1.17** | **-3.40** | **2.23** | σ^W^ |  |
| *yxjI* | **-0.95** | **-3.12** | **2.17** | σ^W^ |  |
| *ydjP* | **-1.22** | **-3.34** | **2.12** | σ^W^ |  |
| *yoaG* | **-0.95** | **-3.07** | **2.12** | σ^W^ |  |
| *pstBA* | 1.50 | -0.56 | **2.06** |  |  |
| *pspA* | **-0.76** | **-2.68** | **1.91** | σ^W^ |  |
| *ythP* | **-1.08** | **-2.98** | **1.90** | σ^W^ | ATP transporter (ATP binding protein) |
| S690 | **-1.01** | **-2.90** | **1.89** | σ^W^ |  |
| *ydjG* | **-0.68** | **-2.51** | **1.83** | σ^W^ |  |
| *yqfA* | -0.37 | **-2.11** | **1.75** | σ^W^ |  |
| *ythQ* | **-1.01** | **-2.74** | **1.73** | σ^W^ | ABC transporter |
| S1495 | **-1.19** | **-2.89** | **1.71** | σ^W^ |  |
| *ydjH* | **-0.57** | **-2.27** | **1.70** | σ^W^ |  |
| *pstBB* | 1.41 | -0.29 | **1.70** |  | Phosphate ABC transporter (binding protein) |
| S719 | **-0.80** | **-2.49** | **1.69** | σ^W^ |  |
| *yqfB* | -0.56 | **-2.25** | **1.69** | σ^W^ |  |
| *ybfO* | **-0.78** | **-2.47** | **1.69** | σ^W^ | Similar to erythromycin esterase |
| *fosB* | **-1.35** | **-3.03** | **1.68** | σ^W^ | Fosfomycin resistance |
| *ydbS* | **-0.79** | **-2.46** | **1.67** | σ^W^ |  |
| *pstA* | 1.21 | -0.45 | **1.66** |  | Phosphate ABC transporter (permease) |
| *ydbT* | **-0.88** | **-2.47** | **1.59** | σ^W^ |  |
| *ydjI* | **-0.61** | **-2.17** | **1.57** | σ^W^ |  |
| *pstC* | 1.15 | -0.41 | **1.56** |  | Phosphate ABC transporter (permease) |
| *yfhL* | **-1.00** | **-2.52** | **1.52** | σ^W^ | SdpC resistance |
| *yqeZ* | -0.72 | **-2.21** | **1.50** | σ^W^ |  |
| *yfhM* | **-0.92** | **-2.30** | **1.38** | σ^W^ | Survival of ethanol stress |
| S160 | -0.19 | **-1.56** | **1.37** | σ^W^ |  |
| *yobJ* | **-0.88** | **-2.24** | **1.36** | σ^W^ |  |
| S658 | **-1.18** | **-2.48** | **1.30** | σ^W^ |  |
| *yuaG* | **-1.06** | **-2.33** | **1.27** | σ^W^ | Sporulation (early stage) |
| *yvlA* | **-0.69** | **-1.91** | **1.22** | σ^W^ |  |
| *yvlB* | **-0.67** | **-1.85** | **1.19** | σ^W^ |  |
| *pbpE* | **-1.20** | **-2.33** | **1.13** | σ^W^ | Cell wall synthesis |
| *ysdA* | 0.80 | -0.33 | **1.12** |  |  |

**Table S4B. Genes down-regulated in the *prsW* mutant strain compared to the *sigW* mutant strain.** Changes associated with p-values < 0.05 are indicated in bold.

| **Name** | ***prsW*/WT** | ***sigW*/WT** | ***prsW/sigW*** | **Function** |
| --- | --- | --- | --- | --- |
| *prsW* | **-4.30** | 0.19 | 0.08 | Control of SigW activity |
| *ybbK* | -0.54 | **3.07** | **-3.76** | Opposite of *sigW* |
| *ybbJ* | -0.70 | **2.68** | **-3.29** | Opposite of *sigW* |
| S928 | -0.41 | **2.25** | **-2.84** | Between *mgsR* and *rsbRD* |
| *cotT* | -0.49 | **0.91** | **-1.31** | Spore coat protein |
| S1492 | -0.69 | 0.55 | -0.21 |  |
| S455 | -0.70 | 0.41 | -0.24 |  |
| *ylaF* | **-0.90** | 0.18 | -0.48 |  |
| S760 | -0.52 | 0.51 | -0.55 |  |
| S415 | **-0.86** | 0.14 | -0.30 |  |
